# Predicting patient outcomes with gene-expression biomarkers from colorectal cancer organoids and cell lines

**DOI:** 10.3389/fmolb.2025.1531175

**Published:** 2025-01-15

**Authors:** Alexandra Razumovskaya, Mariia Silkina, Andrey Poloznikov, Timur Kulagin, Maria Raigorodskaya, Nina Gorban, Anna Kudryavtseva, Maria Fedorova, Boris Alekseev, Alexander Tonevitsky, Sergey Nikulin

**Affiliations:** ^1^ Faculty of Biology and Biotechnologies, National Research University Higher School of Economics, Moscow, Russia; ^2^ P. A. Hertsen Moscow Oncology Research Center, Branch of the National Medical Research Radiological Center, Ministry of Health of the Russian Federation, Moscow, Russia; ^3^ Shemyakin-Ovchinnikov Institute of Bioorganic Chemistry, Russian Academy of Sciences, Moscow, Russia; ^4^ Central Clinical Hospital with Polyclinic, Administration of the President of the Russian Federation, Moscow, Russia; ^5^ Engelhardt Institute of Molecular Biology, Russian Academy of Sciences, Moscow, Russia; ^6^ Art Photonics GmbH, Berlin, Germany; ^7^ Dmitry Rogachev National Medical Research Center of Pediatric Hematology, Oncology and Immunology, Ministry of Health of the Russian Federation, Moscow, Russia

**Keywords:** colorectal cancer, organoids, chemotherapy, drug resistance, response prediction, transcriptomic gene signature

## Abstract

**Introduction:**

Colorectal cancer (CRC) is characterized by an extremely high mortality rate, mainly caused by the high metastatic potential of this type of cancer. To date, chemotherapy remains the backbone of the treatment of metastatic colorectal cancer. Three main chemotherapeutic drugs used for the treatment of metastatic colorectal cancer are 5-fluorouracil, oxaliplatin and irinotecan which is metabolized to an active compound SN-38. The main goal of this study was to find the genes connected to the resistance to the aforementioned drugs and to construct a predictive gene expression-based classifier to separate responders and non-responders.

**Methods:**

In this study, we analyzed gene expression profiles of seven patient-derived CRC organoids and performed correlation analyses between gene expression and IC50 values for the three standard-of-care chemotherapeutic drugs. We also included in the study publicly available datasets of colorectal cancer cell lines, thus combining two different *in vitro* models relevant to cancer research. Logistic regression was used to build gene expression-based classifiers for metastatic Stage IV and non-metastatic Stage II/III CRC patients. Prognostic performance was evaluated through Kaplan-Meier survival analysis and log-rank tests, while independent prognostic significance was assessed using multivariate Cox proportional hazards modeling.

**Results:**

A small set of genes showed consistent correlation with resistance to chemotherapy across different datasets. While some genes were previously implicated in cancer prognosis and drug response, several were linked to drug resistance for the first time. The resulting gene expression signatures successfully stratified Stage II/III and Stage IV CRC patients, with potential clinical utility for improving treatment outcomes after further validation.

**Discussion:**

This study highlights the advantages of integrating diverse experimental models, such as organoids and cell lines, to identify novel prognostic biomarkers and enhance the understanding of chemotherapy resistance in CRC.

## 1 Introduction

Colorectal cancer (CRC) is one of the most prevalent and deadly cancers worldwide, accounting for approximately 1.9 million new cases and over 900,000 deaths each year (Bray et al., 2024). It ranks as the third most common cancer and the second leading cause of cancer-related mortality globally (Bray et al., 2024). The high mortality rate from CRC is largely driven by metastasis, which occurs in a significant proportion of patients. According to statistics, approximately 20% of patients with newly diagnosed CRC present with synchronous metastases, primarily affecting the liver (Cervantes et al., 2023). Furthermore, up to 50% of patients initially diagnosed with localized disease will develop metastases following treatment of initially localized primary tumor (Cervantes et al., 2023). Despite advancements in treatment, including surgery, chemotherapy, and targeted therapies, achieving long-term survival in metastatic CRC remains a significant challenge (Ciardiello et al., 2022). Therefore, it is crucial to develop not only new treatments for metastatic colorectal cancer but also strategies to prevent recurrence, most of which manifest as distant metastases (Haria et al., 2021).

In clinical practice, the assessment of colorectal cancer recurrence risk and therapy selection relies on well-established markers such as TNM staging, MSI status, and mutations in KRAS, NRAS, and BRAF, which are routinely used to guide treatment decisions (Koncina et al., 2020). In addition, emerging gene expression signatures, such as the Oncotype DX Colon and Consensus Molecular Subtypes (CMS), are being integrated into clinical settings to personalize therapy, predict recurrence, and optimize patient outcomes (Ahluwalia et al., 2021). Current molecular biomarkers, such as mutations and expression signatures, mainly reflect the biological characteristics of tumors rather than their sensitivity to therapy. While some of them are useful for guiding targeted and immunotherapy, neither molecular nor clinical markers available today can reliably predict responses to chemotherapy. As chemotherapy remains a cornerstone of colorectal cancer treatment, the limitations of existing biomarkers underscore the need for developing new tools to enhance personalized treatment strategies.

There has been growing interest in testing chemotherapeutic and targeted agents directly on CRC organoids, which accurately replicate the histological and molecular characteristics of the original tumors (van de Wetering et al., 2015; Su et al., 2023). This method offers a personalized approach by allowing precise evaluations of how individual tumors respond to specific drugs, potentially improving treatment outcomes through tailored therapies based on each patient’s unique cancer profile. Currently, this approach is primarily applied to metastatic cancer, where it is easier to compare *in vitro* results with clinical responses (Ooft et al., 2019; Narasimhan et al., 2020; Tan et al., 2023). However, the method has several limitations, including high costs, labor-intensive processes, and challenges in successfully establishing organoid cultures. Furthermore, clinical validation remains limited.

An intriguing alternative is to combine molecular and culturing approaches: identifying drug sensitivity markers from a small set of organoid cultures and applying these markers directly to tumor tissues. This method eliminates the need for extensive organoid culture, making the process faster and more cost-effective. Notably, this combined approach has already been successfully implemented for pancreatic cancer (Tiriac et al., 2018), gastric cancer (Vistoso Monreal et al., 2024) and biliary tract cancer (Ren et al., 2023).

In this study, we expanded our collection of CRC organoids to identify correlations between gene expression and IC50 values for three commonly used CRC drugs: 5-fluorouracil (5-FU), oxaliplatin, and SN-38 (the active metabolite of irinotecan). To ensure robust results, we integrated our organoid data with publicly available data on CRC cell lines, thus bridging two distinct *in vitro* models currently available for cancer research and validating the results of each experiment. As a result, we identified genes whose expression consistently correlates with the response to standard chemotherapeutic drugs. These genes could serve as potential new targets to overcome drug resistance and warrant further investigation. Additionally, using publicly available transcriptomic datasets, we demonstrated that some of these genes possess prognostic value for both early- and late-stage CRC. Gene expression signatures based on these identified genes were shown to effectively predict patient outcomes, underscoring their potential for further translational research. A general flow chart of this study is presented in Supplementary Figure S1.

## 2 Materials and methods

### 2.1 Primary patient material and organoid culture

In this work 4 new cultures of colorectal cancer patient-derived organoids (CRC PDOs) were established from resected metastatic tissue as described previously (Nikulin et al., 2020; Poloznikov et al., 2021). Initial material was obtained from 3 distinct patients P4, P5 and P6 and 2 different metastases from P6 were used for two CRC PDOs P6(1) and P6(2). The generation of CRC PDOs P1, P2 and P3 was described previously (Poloznikov et al., 2021). The main clinical parameters of the patients included in the study are summarized in Supplementary Table S1. The study was approved by the local ethics committee.

The tissue sample was obtained during the examination of the surgically resected tissue block by a qualified pathologist who identified the resected tissue as a metastasis. Tissue was cut into small fragments and placed immediately into MACS tissue storage solution (Miltenyi Biotec, Germany). The sample was stored for several hours at 4°C. Then, the tissue fragments were transferred into a tube for tissue homogenization (gentleMACS C Tube, Miltenyi Biotec, Germany), and dissociated with gentleMACS Octo Dissociator (Miltenyi Biotec, Germany) according to the manufacturer’s instructions. After the end of the program, the resulting suspension was centrifuged at 300 g for 10 min. The supernatant was removed, and the pellet was resuspended in 10 mL of DPBS (Thermo Fisher Scientific, United States of America). Then, the suspension was recentrifuged with the same parameters, the supernatant was also removed, and the pellet was resuspended in DMEM/F-12 culture medium (Thermo Fisher Scientific, United States of America). Then, the tube with the suspension was placed on ice, and the suspension was mixed with Matrigel Growth Factor Reduced (GFR) Basement Membrane Matrix (Corning, United States of America) in the ratio 1:2. Then, 50 μL drops of the resulting suspension in the extracellular matrix were transferred into the wells of a 24-well culture plate (TPP, Switzerland) and placed into a cell culture incubator (37°C, 5% CO_2_) for 20 min for solidification of the gel. Then, 750 μL of complete cell culture medium was added to each well, and the plate was incubated in a cell culture incubator. The recipe of the complete cell culture medium for CRC organoids was described earlier (Poloznikov et al., 2021). The cell culture medium was replaced every 48 h. Cells were inspected visually by an inverted Axio Observer Z1 microscope (Carl Zeiss, Germany). Organoids were subcultured (1:5 dilution) every 2 weeks with the help of TrypLE Express (Thermo Fisher Scientific, United States of America).

### 2.2 Histology

Fragments of the original tumor tissue were fixed in 10% neutral buffered formalin (overnight at room temperature) and embedded into paraffin. Formalin-fixed samples of tumor organoids were covered with Histogel (Thermo Fisher Scientific, United States of America) and then embedded into paraffin. Serial sections with a thickness of 4 μm were cut and then were routinely stained with hematoxylin-eosin and examined by light microscopy.

### 2.3 Drug test

Organoids were diluted in Matrigel GFR Basement Membrane Matrix (Corning, United States of America) and seeded into 96-well plates (TPP, Switzerland) (50 organoids per well). After solidification of the gel, 100 ul of complete cell culture medium was added into each well. After 24 h, the organoid culture medium was replaced with the control medium or the medium containing single standard-of-care (SoC) drugs. Stock solutions of 5-FU and SN-38 were prepared in DMSO; stock solution of Oxaliplatin was prepared in water (Hall et al., 2014). Then CRC organoids were incubated in a cell culture incubator (37°C, 5% CO_2_) for 72 h, and the relative number of viable cells was measured with MTS assay (Promega, United States of America). Each experiment was performed in triplicates. An R package “drc” was used to fit dose-response curves and to determine the half-maximal inhibitory concentration IC50 (Ritz et al., 2015). To confirm the data on sensitivity and resistance, a separate experiment was additionally conducted on two organoid cultures P1 and P3. The size changes of the obtained organoids under conditions similar to the MTS test were assessed using microphotographs obtained by an inverted microscope PrimoVert (Carl Zeiss, Germany).

### 2.4 RNA sequencing

Organoids were lysed with the QIAzol Lysis Reagent (Qiagen, Germany). The lysates were stored at −80°C before RNA isolation. RNA isolation was performed using miRNeasy Micro Kit (Qiagen, Germany) according to the manufacturer’s protocol. Nanodrop ND-1000 (Thermo Fisher Scientific, United States of America) was used to assess the quantity and purity of the extracted RNA. Total RNA samples were also QC-checked using an Agilent 2100 Bioanalyzer (Agilent Technologies, United States of America). For each CRC PDO, three independently obtained samples of RNA were used.

Libraries for mRNA sequencing were prepared from total RNA samples using the Illumina Stranded mRNA Library Prep Kit (Illumina, United States of America). Each sample was sequenced on the NextSeq 550 (Illumina, United States of America) to generate paired-end 75-nucleotide reads.

The quality of FASTQ files was assessed with FastQC v0.11.9 (Babraham Bioinformatics, United Kingdom) and multiQC v1.9 (Ewels et al., 2016). The adapters were trimmed with fastp 0.21.1 (Chen et al., 2018). The trimmed mRNA-seq reads were mapped on the reference human genome GENCODE release 37 (GENCODE GRCh38. primary assembly) with STAR 2.7.7a (Dobin et al., 2013). GENCODE release 37 genome annotation (gencode.v37. primary assembly. annotation) (Frankish et al., 2019) was used to generate the count matrix with the featureCount tool from ssubread-2.0.1 aligner (Liao et al., 2013; 2014).

RNA-seq data generated in this study were deposited into the Gene Expression Omnibus database under accession GSE251958 (https://www.ncbi.nlm.nih.gov/geo/query/acc.cgi?acc=GSE251958).

### 2.5 Gene expression and correlation analysis

This study utilized data on the sensitivity of colorectal cancer cell lines and organoids to SoC chemotherapeutic drugs, as well as RNA expression data. The analysis of cell lines was based on publicly available data from the Genomics of Drug Sensitivity in Cancer (GDSC) (Yang et al., 2012) and the Cancer Cell Line Encyclopedia (CCLE) (Barretina et al., 2012). Sensitivity data for the cell lines to SoC drugs were available across several datasets. For 5-FU, both GDSC1 and GDSC2 datasets were used. Oxaliplatin sensitivity data came from two independent repeats within the GDSC2 dataset, labeled GDSC2 and GDSC2_2. SN-38 data was only available in the GDSC1 dataset. All cell line data were downloaded from the DepMap portal https://depmap.org/portal (Tsherniak et al., 2017).

Gene expression analysis was performed using DESeq2 v1.28.1 (Love et al., 2014). A regularized logarithm transformation of the count data was applied before conducting correlation analysis (Love et al., 2014). Spearman correlation coefficients were calculated for all genes in relation to the IC50 values of each chemotherapeutic drug. Correlations were considered significant if the absolute value of the Spearman correlation coefficient exceeded 0.3 and the p-value was less than 0.05. The threshold for the Spearman correlation coefficient was selected based on prior studies (Shee et al., 2019; Ioannidis et al., 2020) and the understanding that drug response is a complex and multifactorial biological process. In this context, the influence of any single gene is inherently limited. Thus, even modest correlations with a Spearman coefficient of ±0.3 are considered meaningful, particularly when they are reproducible across independent datasets.

An intersection analysis was subsequently conducted to identify genes with significant co-directional correlations across the available datasets (GDSC1, GDSC2, and ORGANOIDS). This resulted in a subset of genes consistently correlated across all datasets.

### 2.6 Pathway analysis

Following the intersection analysis, genes common across all tested datasets were selected for further investigation. For each drug, a list of genes was compiled and categorized based on the direction of their correlation. This gene list was then used as the basis for pathway enrichment analysis.

The enrichment analysis focused on signaling pathways, using an overrepresentation approach. This was performed with the “*enrichPathway”* function from the ReactomePA package, applying the default settings (Yu and He, 2016).

### 2.7 Construction of the gene expression classifier

We used the subset of genes consistently correlated across all datasets to develop prognostic classifiers for both metastatic and non-metastatic colorectal cancer. For late-stage metastatic CRC, our goal was to predict overall survival (OS) status within 5 years, while for early-stage non-metastatic CRC, we aimed to predict relapse-free survival (RFS) status.

For metastatic CRC, we utilized the publicly available dataset GSE159216 from the Gene Expression Omnibus (GEO) (Eide et al., 2021; Moosavi et al., 2021). This dataset contains transcriptomic profiles from metastatic liver tissue of 171 CRC patients. Patients who died within 5 years were categorized as having an unfavorable prognosis, while a favorable prognosis was assigned only if the follow-up period was at least 5 years and the patient was alive. A subset of patients with available prognostic information was split into training (112 patients) and testing (47 patients) datasets. For non-metastatic CRC we used data from the GSE39582 dataset (Marisa et al., 2013). Initially, we selected only the patients who had II or III stage of the disease and who were treated with chemotherapy. This filter resulted in the whole treated patient dataset with 210 patients. Then we defined the status of the patients as favorable in case of absence of relapse and time of follow-up more or equal to 5 years and unfavorable in case of developing of relapse within 5 years. The subset of patients with available prognostic status was split to construct train (112 patients) and test (47 patients) datasets. We also evaluated the performance of the best constructed classifier on a group of untreated patients at the same disease stages from the same dataset. The microarray data were normalized using the Robust Multiarray Average (RMA) method, implemented in the *“affy”* package in R (Gautier et al., 2004).

The classifier constructed for Stage II/III CRC patients was further validated using the mRNA-seq dataset E-MTAB-12862 from ArrayExpress, with a training cohort of 441 patients and a testing cohort of 189 patients (Nunes et al., 2024). Raw count data derived from STAR alignment were normalized using the trimmed mean of M values (TMM) method followed by log-transformed counts per million (logCPM), as implemented in the *“edgeR”* package (Robinson et al., 2010; McCarthy et al., 2012; Chen et al., 2016).

To construct gene expression-based classifiers, we employed a sequential algorithm using logistic regression, implemented with the *“glm”* function in R. The classifiers were trained exclusively on the training datasets. Initially, models were built using a single gene from the list of consistently correlated genes, with additional genes added stepwise, up to a total of 12 genes. At each step, models were retained if the newly added gene was at least marginally significant (p-value <0.15) and all other genes were significant (p-value <0.05). Additionally, we conducted repeated 3-fold cross-validation at each step, selecting the top 50% of models based on average ROC-AUC values.

Survival analysis for groups predicted by the best classifier (with a threshold set at 0.5) was performed using the Kaplan-Meier method and log-rank test. To assess whether the gene expression-based model served as an independent prognostic factor when accounting for other clinically relevant variables, we used a multivariate Cox proportional hazards model. All analyses were conducted in R using the *“survival”* and *“survminer”* packages.

### 2.8 Analysis of biological mechanisms underlying prognostic classifications

To investigate the differences in molecular pathways and immune response-related pathways between favorable and unfavorable prognosis groups in CRC at Stages II-III and IV, we employed the clusterProfiler package along with the KEGG (Kyoto Encyclopedia of Genes and Genomes) and GO (Gene Ontology) databases (Ashburner et al., 2000; Kanehisa and Goto, 2000; Yu et al., 2012). Differentially expressed genes were identified using the *“limma”* package, and enrichment analyses for KEGG pathways and GO biological processes were performed with a significance threshold of an adjusted p-value <0.05 (Ritchie et al., 2015).

To examine immune and stromal differences between prognosis groups, the ESTIMATE algorithm was applied (Yoshihara et al., 2013). Preprocessed and normalized gene expression data were used to calculate immune and stromal scores, as well as tumor purity estimates. A two-sample *t*-test was conducted to evaluate the statistical significance of score differences between prognosis groups.

For detailed immune cell population analysis, the xCell algorithm was utilized (Aran et al., 2017). Normalized gene expression data were input into xCell to estimate the relative abundances of various immune and stromal cell types. Differences in cell type proportions between prognosis groups were assessed using a *t*-test, with adjusted p-values calculated via the Benjamini–Hochberg (BH) method to account for multiple comparisons. Results were visualized using boxplots, displaying the distribution of each cell type stratified by prognosis group, providing insights into the immune microenvironment of CRC in relation to patient outcomes.

## 3 Results

### 3.1 Morphological similarities between patient-derived tumor organoids and corresponding clinical tumors

**FIGURE 1:**
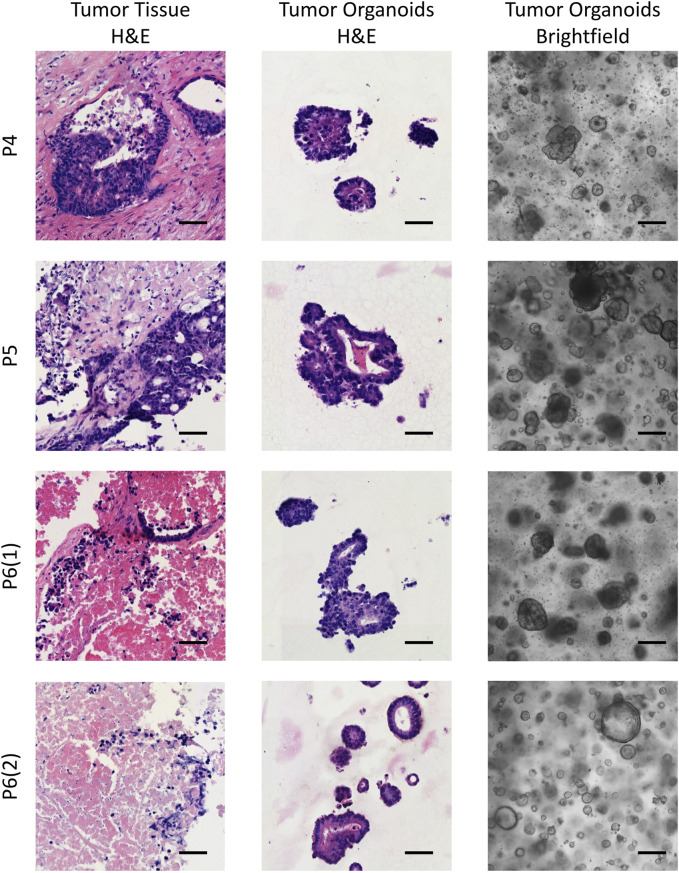
Brightfield images of the established CRC PDOs alongside comparative histological analysis of H&E-stained slides from the initial tissue and organoids. P4–P6 refers to patient numbers. Scale bars represent 200 μm in the brightfield images and 50 μm in the H&E images.

Patient-derived colorectal cancer organoids (CRC PDOs) were generated from digested tumor tissue, embedded in Matrigel, and cultured for up to 3 weeks without passaging. After subculturing, significant cell growth was observed as early as the second day post-seeding. To verify that the organoids consisted of colorectal cancer cells rather than normal epithelial cells, H&E histological analysis was performed on the established CRC PDOs. An experienced pathologist compared the morphology of the organoids to that of the primary tumors, confirming that all organoid cultures were composed of malignant cells (Figure 1; Supplementary Figure S2).

### 3.2 Response of CRC patient-derived organoids (PDOs) to standard-of-care chemotherapeutic drugs

**FIGURE 2:**
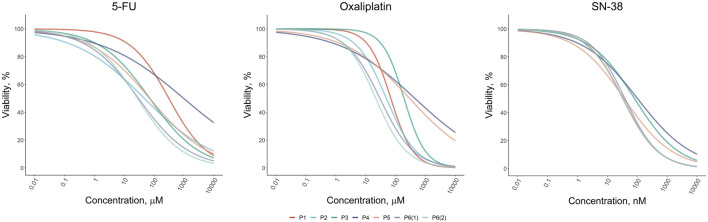
Dose-response results of the drug test for standard-of-care drugs (5-FU, Oxaliplatin, and SN-38) on colorectal cancer organoids derived from patients P1–P6.

Our results revealed that the response to standard chemotherapeutic drugs varied significantly across the tumor organoid cultures (Figure 2). Organoids from patients P1 and P4 exhibited the highest resistance to 5-FU, with IC50 values of 313 µM and 984 μM, respectively, which were 6 and 20 times higher than the average IC50 values for other patients. For oxaliplatin, organoids from patients P3, P4, and P5 showed the greatest resistance, with IC50 values ranging from 190 to 440 μM, compared to 20–70 µM for other patients. Interestingly, organoids from P3 and P4 also displayed the highest resistance to SN-38, although the variation in SN-38 sensitivity across organoids was less pronounced compared to the other drugs. These viability assays allowed us to identify organoid cultures with differing levels of sensitivity and resistance, highlighting potential variations in drug responsiveness among patients. The results of the drug sensitivity and resistance tests were further validated through micrographs of the two organoid cultures, P1 and P3, taken after 72-h treatment with the drugs, as well as under control conditions (Supplementary Figure S3). These visual observations confirmed that P1 exhibited greater resistance to 5-FU compared to P3, while P3 showed higher resistance to Oxaliplatin and SN-38 compared to P1. These findings were fully consistent with the results obtained from the MTS assay, further supporting the robustness of our observations.

### 3.3 Correlation analysis of gene expression with resistance to standard-of-care drugs

**FIGURE 3:**
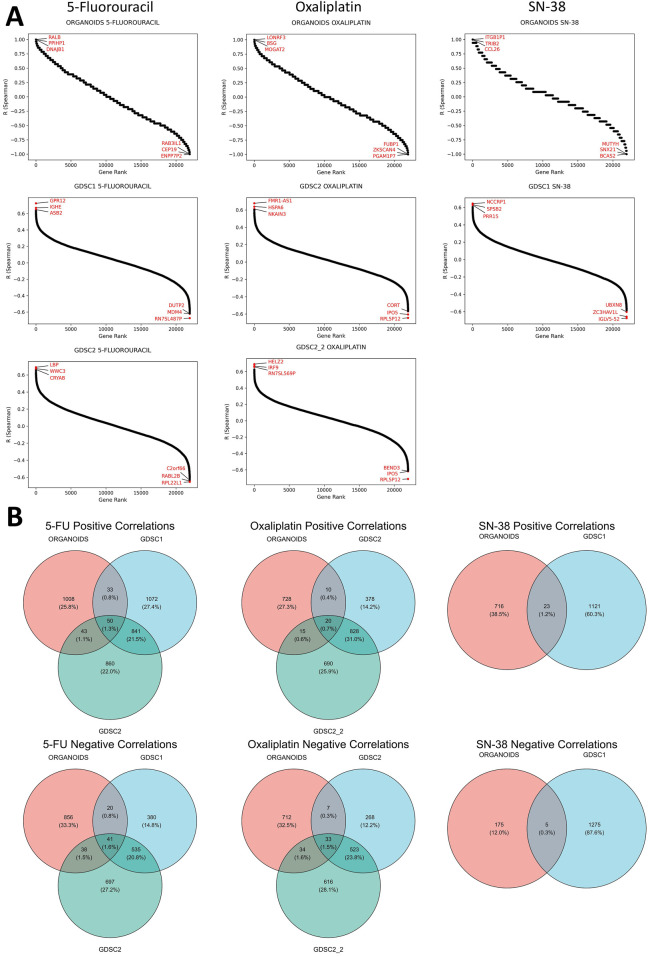
Results of the correlational analysis between gene expression levels and IC50 values in established CRC patient-derived organoids and cell lines. **(A)** Ranked Spearman correlation coefficients displaying the relationship between gene expression levels and IC50 values of standard-of-care drugs across different datasets, with genes showing the highest correlation coefficients marked in the plots. **(B)** Venn diagrams showing the overlap of significantly correlated genes for each SOC drug across the different datasets.

Correlational analysis of gene expression and IC50 values for established CRC PDOs and CRC cell lines from publicly available datasets (GDSC1, GDSC2, GDSC2_2) revealed a large number of genes whose expression was positively or negatively correlated with sensitivity to standard chemotherapy drugs (Figure 3). The number of genes significantly correlated with drug sensitivity in a single direction for one dataset ranged from a few hundred to nearly two thousand. Notably, the reproducibility of the correlations between the CRC cell line datasets was moderate. Furthermore, the number of genes consistently correlated with drug sensitivity across both CRC cell lines and organoids was very limited. For 5-FU, a total of 91 genes were consistently correlated (50 positively and 41 negatively), while for oxaliplatin, only 53 genes were identified (20 positively and 33 negatively). Interestingly, SN-38 had the smallest number of consistently correlated genes across datasets, with 23 genes showing positive correlations and 5 showing negative correlations; however, this analysis involved only two datasets (ORGANOIDS and GDSC1). The complete list of intersecting genes is provided in the Supplementary Tables S2–S4. Selected genes associated with resistance to standard chemotherapy will be discussed in detail in the Discussion section.

We also aimed to identify genes whose expression correlated with sensitivity to more than one SoC drug. The analysis revealed that the number of such genes was extremely limited, with no genes found in the triple intersection of the Venn diagrams for all three SoC drugs (Supplementary Figure S4). Furthermore, no genes showed significant correlations in opposite directions for any pair of tested SoC drugs, indicating no contradictory findings regarding chemotherapy sensitivity (Supplementary Figure S5). However, common genes were identified between 5-FU and oxaliplatin: one gene (*AVPI1*) showed a positive correlation, while four genes (*DHX33, LYRM2, RANGRF, WRAP53*) exhibited negative correlations with IC50 values for both drugs across all datasets. These genes will be discussed in detail in the Discussion section.

### 3.4 Pathway analysis

Several significantly enriched pathways were identified based on genes whose expression consistently correlated with drug sensitivity (Supplementary Table S5). For 5-FU, the pathways “Iron Uptake and Transport” and “Transferrin Endocytosis and Recycling” were activated in more resistant cells. In oxaliplatin-resistant cells, pathways such as “Rab Regulation of Trafficking” and “RAB GEFs exchange GTP for GDP on RABs” were identified. In contrast, oxaliplatin-sensitive cells showed activation of pathways like “Transport of Mature mRNA derived from an Intron-Containing Transcript”, “Transport of Mature Transcript to Cytoplasm”, and “Processing of Capped Intron-Containing Pre-mRNA”, indicating enhanced nuclear pore complex transport. In SN-38-sensitive cells, pathways associated with taste receptors, which belong to the GPCR transmembrane protein superfamily, were activated. These pathways include “Sensory Perception of Taste”, “Sensory Perception of Sweet, Bitter, and Umami (Glutamate) Taste”, and “GPCR Ligand Binding”. The role of some of these pathways in cancer progression will be further explored in the Discussion section.

### 3.5 Predictive 12-gene classifier assessing overall survival of patients with metastatic CRC

Using genes whose expression is consistently associated with resistance to the three tested standard-of-care drugs, we developed a series of classifiers to predict OS status in patients with metastatic Stage IV colorectal cancer. Gene expression data from liver metastasis samples (GSE159216) were used, with the dataset split into training and test sets. Classifiers were constructed using logistic regression models incorporating 12 genes, with over 6 million classifiers generated based on various gene combinations. All classifiers were trained and cross-validated on the training dataset, and their performance was subsequently evaluated on the test dataset. To identify the most stable classifier, we selected models with ROC-AUC values exceeding 0.85 on both training and test datasets, and the best classifier was chosen based on the highest ROC-AUC value achieved during cross-validation. This approach allows for the identification of a stable gene expression signature most suitable for further validation. A list of genes and model coefficients for the optimal signature is provided in the Supplementary Table S6, with select genes discussed in the Discussion section.

**FIGURE 4:**
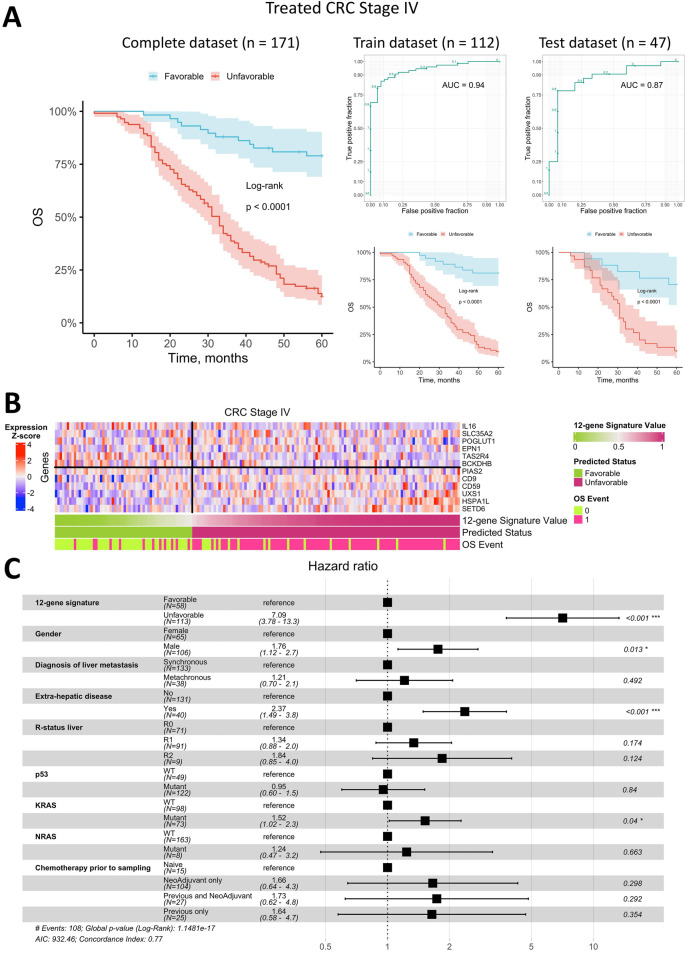
Prediction of overall survival for patients with metastatic Stage IV CRC using a 12-gene expression signature. **(A)** Kaplan-Meier plots for the training, test, and complete (including censored data) datasets, along with ROC curves for the training and test datasets. **(B)** Heatmap of gene expression in metastatic tumor tissue for genes included in the 12-gene signature, with classifier predictions and actual OS status included. **(C)** Forest plot displaying the results of a multivariate Cox regression model, identifying potential risk factors for overall survival in patients with metastatic Stage IV CRC.

The best classifier achieved an AUC of 0.94 on the training dataset and 0.87 on the test dataset (Figure 4A). Kaplan-Meier analysis for the training, test, and complete datasets (the latter also includes patients with uncertain status) demonstrated a significantly better overall survival for patients predicted to have a favorable OS status (log-rank p < 0.0001). Approximately two-thirds of metastatic CRC patients were classified as having an unfavorable prognosis, with only one-third predicted to have a favorable prognosis. The 5-year survival rate for patients with a favorable prognosis exceeded 75%, which is uncommon in metastatic CRC.

The heatmap of gene expression for the 12-gene signature (Figure 4B) indicates that robust OS status prediction cannot rely on any single gene alone, despite trends of higher or lower expression in metastatic tissue samples from patients with differing prognoses. However, the combined expression of these genes produced a highly robust classifier with a low error rate.

To assess whether the OS status predicted by the 12-gene expression signature was an independent prognostic factor, we performed a Cox multivariate analysis alongside available clinical parameters and visualized the results in a forest plot (Figure 4C). The predicted OS status was confirmed as an independent prognostic factor, exhibiting the highest HR of 7.1 (95% CI: 3.8–13.3). The next strongest independent factors were the presence of extrahepatic metastases (HR = 2.4, 95% CI 1.5–3.8), gender (HR = 1.8, 95% CI 1.1–2.7), and KRAS mutations (HR = 1.5, 95% CI 1.0–2.3). All other factors were insignificant in the multivariate analysis.

### 3.6 Predictive 12-gene classifier assessing relapse-free survival of patients with stage II/III CRC

Similar to metastatic Stage IV CRC, we searched classifiers based on genes whose expression is consistently associated with resistance to the three standard-of-care drugs in non-metastatic Stage II/III CRC, to predict RFS status. For this analysis, we used the publicly available dataset GSE39582, which includes gene expression data from primary colorectal tumors. We first focused on patients treated with adjuvant chemotherapy, as our genes of interest relate to response to SoC chemotherapy. The dataset was split into training and test sets. We included 12 genes in the classifiers, as in the metastatic CRC analysis, and generated approximately 123,000 classifiers in total—a much smaller number than for metastatic CRC. For Stage II/III CRC, no classifiers reached a ROC-AUC value over 0.85 on both training and test datasets, so we lowered the threshold to 0.75. As with metastatic CRC, the best classifier was selected based on the highest ROC-AUC value obtained during cross-validation. This optimal classifier was then tested on patients who had not received adjuvant chemotherapy. The genes and coefficients for the best gene expression signature for Stage II/III CRC are available in Supplementary Table S7, with selected genes discussed in the Discussion section.

**FIGURE 5:**
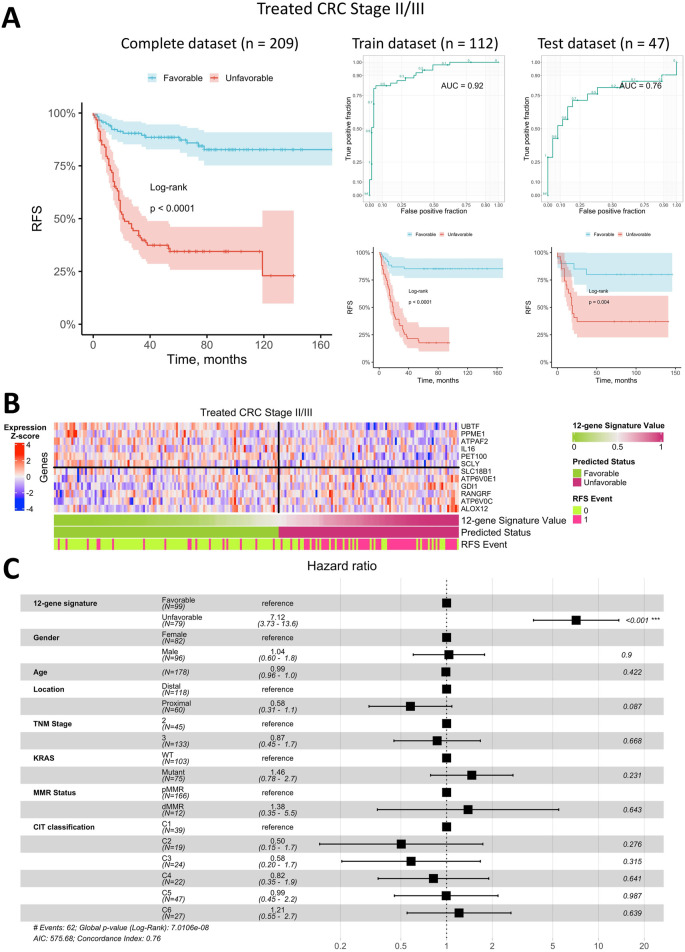
Prediction of relapse-free survival for Stage II/III CRC patients treated with adjuvant chemotherapy using a 12-gene expression signature. **(A)** Kaplan-Meier plots for the training, test, and complete (including censored data) datasets, alongside ROC curves for the training and test datasets. **(B)** Heatmap of gene expression in primary tumor tissue for genes in the 12-gene signature, including classifier predictions and actual RFS status. **(C)** Forest plot showing results of a multivariate Cox regression model, identifying potential risk factors for RFS in Stage II/III CRC patients treated with adjuvant chemotherapy.

Overall, the best classifier’s AUC for treated Stage II/III CRC was lower than for metastatic CRC, at 0.92 on the training dataset and 0.76 on the test dataset (Figure 5A). Kaplan-Meier analysis of the training, test, and complete datasets (the latter includes patients with uncertain status) for Stage II/III CRC patients treated with adjuvant chemotherapy indicated a significantly better prognosis for patients predicted to have a favorable RFS status (log-rank p < 0.0001 for training and complete datasets; log-rank p = 0.004 for the test dataset). Approximately 56% of treated Stage II/III CRC patients were classified as having a favorable prognosis, while 44% were classified as unfavorable. Notably, the 5-year survival rate for patients with an unfavorable prognosis was approximately 35%, which is notably low for early-stage CRC.

The heatmap of gene expression for the 12-gene signature (Figure 5B) demonstrates that robust RFS status prediction in Stage II/III CRC patients treated with adjuvant chemotherapy cannot rely on any single gene alone, though some genes showed distinct expression trends in primary tumor samples from patients with different prognoses. However, the classifier combining all 12 genes was robust, with a low error rate similar to that observed in metastatic CRC.

To assess whether the predicted RFS status based on the 12-gene expression signature is an independent prognostic factor, we performed a Cox multivariate analysis incorporating available clinical parameters and visualized the results in a forest plot (Figure 5C). This analysis indicated that the predicted status was the only independent prognostic factor with HR of 7.1 (95% CI 3.7–13.6). All other factors, including the TNM stage and molecular CIT subtype (Marisa et al., 2013), were found to be insignificant in the multivariate analysis.

**FIGURE 6:**
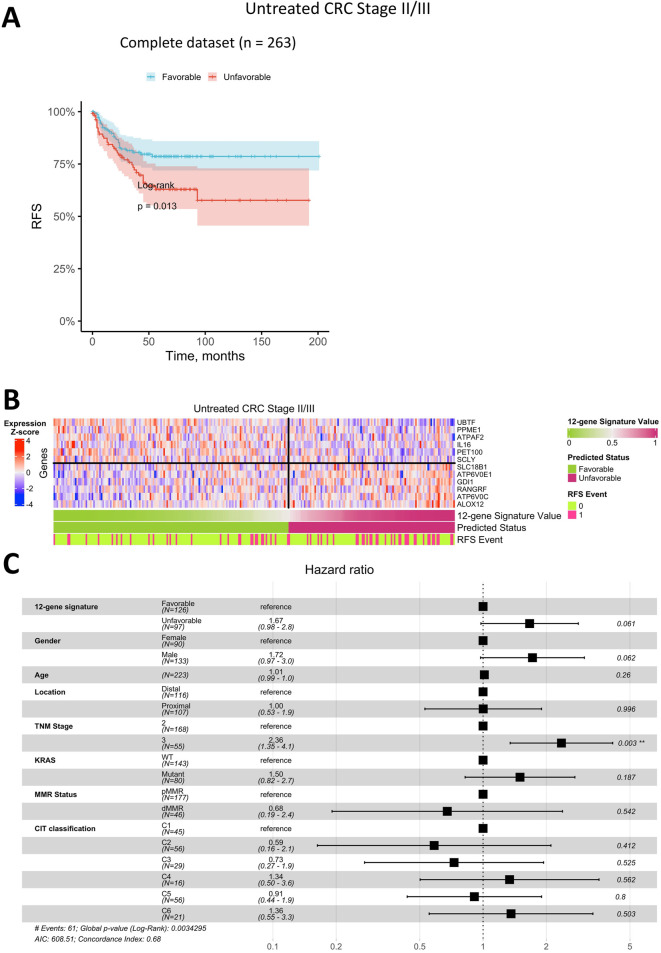
Prediction of relapse-free survival for Stage II/III CRC patients untreated with adjuvant chemotherapy using a 12-gene expression signature. **(A)** Kaplan-Meier plot for the complete (including censored data) dataset. **(B)** Heatmap of gene expression in primary tumor tissue for genes in the 12-gene signature, including classifier predictions and actual RFS status. **(C)** Forest plot showing results of a multivariate Cox regression model, identifying potential risk factors for RFS in Stage II/III CRC patients untreated with adjuvant chemotherapy.

We next evaluated whether the best classifier, developed using data from Stage II/III CRC patients treated with adjuvant chemotherapy, could predict RFS status in patients who did not receive adjuvant chemotherapy. Kaplan-Meier analysis (Figure 6A) indicated that patients with a predicted favorable prognosis had a higher RFS rate (log-rank p = 0.013). However, the survival difference between patients with favorable and unfavorable prognoses was notably smaller than that observed in the adjuvant-treated group. Among Stage II/III CRC patients untreated with adjuvant chemotherapy, approximately 59% were classified as having a favorable prognosis, and 41% as unfavorable, which is comparable to the distribution seen in the adjuvant-treated cohort. Additional validation was conducted using the RNA-seq dataset of Stage II/III CRC patients (Nunes et al., 2024). Kaplan-Meier analysis (Supplementary Figure S6) revealed that patients with a predicted favorable prognosis had a relapse-free survival rate exceeding 75%, compared to less than 50% in patients with an unfavorable prognosis (log-rank p = 0.003 in the training dataset and log-rank p = 0.049 in the test dataset). These findings further support the classifier’s robustness and its ability to reliably distinguish between favorable and unfavorable prognosis groups across independent datasets.

Similar to the treated cohort, the heatmap of gene expression for the 12-gene signature in untreated patients (Figure 6B) demonstrated that robust RFS prediction would not be feasible based on the expression of a single gene. Although certain genes exhibited clear expression patterns associated with prognosis, the combined 12-gene classifier was not as robust, showing a higher error rate in this group.

In Cox multivariate analysis of clinical parameters for untreated Stage II/III CRC patients (Figure 6C), the RFS status predicted by the 12-gene signature was not an independent prognostic factor (p > 0.05). In this group, the only significant independent prognostic factor was TNM Stage, with HR of 2.4 (95% CI 1.4–4.1).

### 3.7 Analysis of biological mechanisms underlying prognostic classifications

To elucidate the biological mechanisms underlying prognostic classifications, we examined differences in molecular pathways, immune response-related pathways, immune scores, stromal scores, and immune cell proportions between favorable and unfavorable prognosis groups. This comprehensive analysis was performed across both early (Stages II/III) and late (Stage IV) stages of CRC to provide a detailed understanding of the factors contributing to patient outcomes.

In Stages II-III, KEGG pathway enrichment analysis revealed significant activation of pathways associated with oxidative phosphorylation, thermogenesis, and extracellular matrix (ECM) organization in the unfavorable prognosis group (Supplementary Table S8). These findings emphasize the pivotal role of enhanced mitochondrial activity and ECM remodeling, both of which are integral to tumor growth and localized invasion. Supporting this, GO analysis highlighted mitochondrial respiratory chain complex assembly and ECM structural organization as key biological processes driving these outcomes (Supplementary Table S9). Together, these results underscore the critical influence of metabolic activity and ECM dynamics in determining the prognosis of early-stage CRC.

In Stage IV tumors, KEGG pathway enrichment analysis identified significant activation of immune-related pathways and pathways associated with cellular stress and proliferation in the unfavorable prognosis group (Supplementary Table S10). Notably, pathways related to infection, such as *Salmonella* infection, were enriched, potentially indicating a link to immune responses or inflammation within the tumor microenvironment. Additional enriched pathways included those involved in cell cycle regulation and apoptosis, aligning with the high proliferative and adaptive capacity that characterizes metastatic cancer (Supplementary Table S11). These findings highlight the complex interplay between immune dynamics, cellular stress, and proliferation in shaping the outcomes of advanced-stage CRC.

In Stages II-III, patients with a favorable prognosis exhibited significantly lower immune (p = 4.6E-02) and stromal scores (p = 2.5E-05) compared to those with an unfavorable prognosis, reflecting a tumor microenvironment with reduced immune infiltration (Supplementary Figure S7). Higher tumor purity was also observed in favorable prognosis cases (p = 4.0E-04), suggesting a less complex and potentially less immunologically active tumor microenvironment. ESTIMATE scores further supported these findings, showing significant differences between prognosis groups (p = 6.4E-04).

Conversely, in Stage IV tumors, no significant differences in immune, stromal, or ESTIMATE scores were observed between the favorable and unfavorable prognosis groups (Supplementary Figure S8). This indicates that the tumor microenvironment in metastatic CRC is consistently immune-enriched, regardless of prognosis.

Immune cell composition analysis showed minimal variations specific to disease stages. In Stages II-III, no significant differences in the proportions of individual immune cell types were identified between prognosis groups (adjusted p-value ≥0.05), suggesting that the differences in the tumor microenvironment are systemic rather than cell-type specific (Supplementary Table S12; Supplementary Figures S9A, 9B). However, the significant differences in overall immune and stromal scores underscore broader changes in the immune landscape between prognosis groups in this case.

In Stage IV tumors, immune cell composition remained consistent across prognosis groups, with no significant differences identified (adjusted p-value ≥0.05) (Supplementary Table S13; Supplementary Figures S10A, 10B). These findings suggest that the immune cell profile in metastatic CRC is stable and does not vary significantly with prognosis.

## 4 Discussion

The treatment of metastatic colorectal cancer remains a significant challenge (Ciardiello et al., 2022). Therefore, developing new therapeutic options for metastatic CRC, along with strategies to prevent recurrence in initially localized disease—often manifesting as distant metastases—continues to be essential (Haria et al., 2021). With an increasing array of treatments available, precision medicine is gaining importance in ensuring that the most effective therapies are selected for each patient. Tumor organoids represent a promising tool for assessing personalized treatment sensitivity. Studies have shown that CRC organoids can reasonably predict clinical responses (Ooft et al., 2019; Narasimhan et al., 2020; Tan et al., 2023). However, several factors currently limit their broader use, including challenges in establishing organoid cultures from tumor tissue, lack of immune or stromal components in the organoid system, high costs, and labor-intensive procedures. Although the direct clinical application of tumor organoids is still under development, organoids serve as highly relevant *in vitro* models (Veninga and Voest, 2021). They reflect many characteristics of the original tumors and offer a valuable platform for identifying novel biomarkers, including those associated with drug resistance (Mir et al., 2016; Ryu et al., 2024).

In this study, we expanded our previously established CRC organoid collection (Poloznikov et al., 2021) to identify robust biomarkers associated with resistance to three standard-of-care chemotherapeutic agents — 5-FU, oxaliplatin, and SN-38 (an active metabolite of CPT-11 or irinotecan), which are key components of systemic CRC treatment. To achieve this, we conducted mRNA sequencing on the organoids and performed cytotoxicity assays to assess drug response, aiming to identify genes with expression levels that correlate with drug resistance. These genes could serve as biomarkers for drug resistance and may also represent therapeutic targets. Even modest correlations with a Spearman coefficient of ±0.3 are meaningful if reproducible, as this ensures reliability in the context of drug response, a complex biological process where the influence of any single gene is often limited. For the first time, in our study we conducted correlational analyses using both CRC organoids and publicly available CRC cell line data to ensure reliable findings. The intersection of gene lists from these datasets allowed us to pinpoint genes whose expression consistently correlates with resistance to each SoC drug. We would like to emphasize that our novel approach of combining two *in vitro* models enabled the identification of new genes previously not associated with drug resistance. Finally, these genes were used to construct gene expression signatures to predict overall survival in Stage IV CRC patients and relapse-free survival in Stage II/III CRC patients.

Our results highlight the limited reproducibility in studies that correlate drug resistance with gene expression, especially when comparing data across different models. Even within comparisons of cancer cell lines alone, reproducibility is modest, and this becomes more pronounced when comparing cell lines to tumor organoids. This may be partly due to varying experimental conditions. Additionally, tumor organoids, as more advanced models, may inherently yield different results than traditional cancer cell lines. However, the extensive use of cancer cell lines in research has demonstrated their utility, underscoring the importance of synthesizing findings from diverse model types to ensure robust, reliable conclusions. In the future, larger studies on CRC organoids may reveal additional genes associated with resistance to commonly used chemotherapeutic agents in CRC treatment—genes that might not be detectable in cell line models.

The first drug we studied, 5-fluorouracil (5-FU), is a cornerstone of chemotherapy for CRC treatment (Kumar et al., 2023). Structurally, 5-FU is a heterocyclic aromatic compound similar to pyrimidines, specifically resembling uracil but with a fluorine atom substituting for hydrogen at the C-5 position. This structural similarity enables 5-FU to be incorporated into RNA and DNA, disrupting nucleoside metabolism and causing cytotoxicity and death in rapidly dividing cells (Noordhuis et al., 2004). In mammalian cells, 5-FU is metabolized into three main active forms: fluorodeoxyuridine triphosphate (FdUTP), fluorouridine triphosphate (FUTP), and fluorodeoxyuridine monophosphate (FdUMP). Among these, FdUMP inhibits thymidylate synthase (TS) by forming a stable complex that blocks the synthesis of deoxythymidine monophosphate (dTMP), an essential nucleotide for DNA replication and repair (Longley et al., 2003).

Currently, numerous mechanisms of resistance to 5-FU have been identified. Some are broadly relevant across anticancer drugs, such as the expression of ABC transporters, while others are more specific to 5-FU, involving enzymes that metabolize the drug (Azwar et al., 2021). In this study, we identified additional genes linked to 5-FU response, with *UXS1* standing out as particularly notable. *UXS1* encodes an enzyme that converts UDP-glucuronic acid to UDP-xylose. Interestingly, previous research has connected this gene to 5-FU resistance in fungal cells (Billmyre et al., 2020). In fungi, mutations and downregulation of *UXS1* lead to the accumulation of UDP-glucuronic acid, which may hinder the formation of toxic 5-FU metabolites or interfere with the inhibition of drug targets, like TS. Consequently, in fungi, *UXS1* expression correlates negatively with 5-FU resistance. However, our findings suggest a different relationship in CRC, where *UXS1* expression is distinctly associated with 5-FU resistance. In our study, *UXS1* expression showed a positive correlation with IC50 values for 5-FU, indicating that higher *UXS1* expression is linked to increased drug resistance. Furthermore, elevated *UXS1* expression significantly increased the risk of death in patients with metastatic CRC. Notably, recent work has demonstrated that cisplatin-resistant lung and breast cancer cells exhibit sensitivity to *UXS1* knockout (Doshi et al., 2023). This underscores the potential of *UXS1* as a novel therapeutic target in overcoming chemoresistance in certain cancers, including CRC.

Our pathway analysis revealed, among others, a pathway for iron uptake and transport. There is now a large body of research showing that resistance to anticancer drugs significantly alters iron metabolism depending on the type of cancer and the drugs used. Since iron is an essential element for cell proliferation, various drug-resistant cells with high proliferation rates often increase iron uptake, thereby increasing its intracellular level (Kazan et al., 2017). Particularly, increased expression of several genes, associated with this pathway, including the V-ATPase subunit genes *ATP6V0C* and *ATP6V0E1*, was linked to heightened resistance to 5-FU. V-ATPase is involved in membrane trafficking, receptor recycling (e.g., transferrin and insulin receptors), and autophagy (Collins and Forgac, 2020). Elevated V-ATPase expression correlates with poor prognosis across many cancer types, and its inhibition presents a potential anticancer strategy (Chen F. et al., 2022). Notably, higher expression of *ATP6V0C* and *ATP6V0E1* was significantly associated with worse prognosis for Stage II/III CRC patients.

In addition, it was revealed that increased expression of the genes *HSPA1L* and *SETD6* was significantly correlated with resistance to 5-FU. Their high expression levels were found to be associated with poor prognosis for metastatic colorectal cancer. Previous research has established that heat shock 70-kDa protein 1-like (*HSPA1L*) is crucial in promoting CRC proliferation via HIF-1α activation and cellular prion protein (PrP^C^) regulation within tumor niches (Lee et al., 2017), and its expression has already been linked to CRC prognosis (Lee et al., 2019; Huang et al., 2024). Similarly, *SETD6* has been implicated as a marker in breast cancer stem cells, and its mutation is associated with increased susceptibility to familial colorectal cancer type X (Martín-Morales et al., 2017; Li et al., 2023). Overall, our study highlights the previously underexplored role of these genes in 5-FU resistance, offering novel insights into their potential as therapeutic targets in CRC.

Conversely, higher expression of certain genes linked to 5-FU response was associated with a significantly improved prognosis in CRC patients. One prominent example is *POGLUT1* (Protein O-Glucosyltransferase 1), whose expression showed a negative correlation with 5-FU IC50 values, suggesting increased sensitivity to the drug. Consistent with this, elevated *POGLUT1* expression significantly lowered the risk of death for metastatic CRC patients. Interestingly, previous studies linked higher POGLUT1 levels to more advanced CRC (Mehboob and Lang, 2021). POGLUT1 expression has been observed to be significantly elevated in colorectal cancer tissues compared to adjacent noncancerous areas, with overexpression linked to advanced TNM stages, lymph node metastasis, and shorter survival times (Fang et al., 2017). Knockdown of *POGLUT1* in CRC cells has been shown to halt proliferation and enhance cell adhesion (Fang et al., 2017). These findings underscore how the role of specific genes in cancer progression and therapeutic response can vary widely, sometimes even showing opposite effects.

Another notable example is *IL16*, where higher expression is associated with significantly improved prognosis. Intriguingly, *IL16* was the only gene included in both gene expression signatures for metastatic Stage IV CRC and early Stage II/III CRC patients. The limited overlap between metastatic and non-metastatic CRC gene signatures can be attributed to the fundamentally distinct characteristics of primary and metastatic tumors. In early-stage CRC, chemotherapy primarily targets a small population of residual cancer cells that have not yet initiated metastasis. In contrast, metastatic cancer involves widely disseminated cells that have already adapted to the tumor microenvironment and developed enhanced resistance to treatment (D’Alterio et al., 2020). Interleukin-16, a multifunctional cytokine, plays a key role in inflammatory diseases and contributes to tumor development and progression (Kovacs, 2001; Richmond et al., 2014). Recent studies have identified single nucleotide polymorphisms (SNPs) in the *IL16* gene as a potential factor in CRC susceptibility. While CRC patients have significantly higher serum IL-16 levels than healthy individuals, no significant association was found between *IL16* polymorphisms and serum IL-16 levels (Gao et al., 2008). In our analysis, *IL16* expression was positively correlated with 5-FU resistance *in vitro*, with higher expression observed in more resistant cells. However, patient data showed an opposite association, where elevated *IL16* expression was linked to a better prognosis. This discrepancy underscores the limitations of directly translating *in vitro* findings, even from advanced models like organoids, into clinical observations. One possible explanation for this opposing effect is that *IL16* is predominantly expressed in immune cells, not epithelial cells. Higher *IL16* expression in tumor tissue may indicate greater immune system activation, which could enhance the patient’s prognosis.

The second key CRC drug explored in this study is oxaliplatin, a third-generation platinum-based medication commonly used in colorectal, gastric, and pancreatic cancers (Mustafa et al., 2024a). Its introduction has notably improved objective response rates and progression-free survival in metastatic CRC patients. Oxaliplatin functions primarily by binding to DNA to create adducts that cause DNA damage, which disrupts replication and transcription processes, ultimately leading to apoptosis and triggering immune responses (Alcindor and Beauger, 2011). Despite these therapeutic benefits, oxaliplatin efficacy is often compromised by tumor resistance, making it essential to explore the molecular mechanisms behind this resistance to optimize treatment strategies. While previous studies have provided insights into the mechanisms of oxaliplatin resistance and its biomarkers (Martinez-Balibrea et al., 2015; Escalante et al., 2021), much remains to be explored. A considerable number of genes associated with oxaliplatin resistance relate closely to its mechanism of action, with DNA repair genes such as *ERCC1*, *ERCC2*, *XRCC1*, and *OGG1* playing essential roles in counteracting oxaliplatin-induced damage. Genes involved in drug transport, such as *ATP7A/B*, *ABCC1/4*, and *ABCB1*, also affect cellular uptake and efflux, impacting drug sensitivity. Additionally, genes tied to apoptosis, including *BCL2*, *BIRC5*, *BAX*, and *XIAP*, are involved in oxaliplatin resistance.

Through our correlation analysis, we identified several genes linked with oxaliplatin resistance in CRC organoids and cell lines that have previously been associated with CRC development or proposed as prognostic biomarkers. Notable examples include *TRIM37* (Hu and Gan, 2017; Zhao et al., 2017), *HNRNPH1* (Takahashi et al., 2020), *MCM4* (Ahluwalia et al., 2019), *RIOK1* (Chen Y. et al., 2022) and *GDI1* (Xie et al., 2021). Moreover, some of these genes demonstrated significant prognostic associations in our study, further underscoring their potential role in CRC progression and response to treatment.

One such gene is *BCKDHB*, which encodes a subunit of branched-chain keto acid (BCKA) dehydrogenase. In our study, higher *BCKDHB* expression was linked to both lower IC50 of oxaliplatin *in vitro* and reduced mortality among metastatic CRC patients. BCKDHB is a critical component of the multimeric enzyme BCKDH, which catalyzes the oxidative decarboxylation of BCKA, producing acyl-CoA derivatives and playing an essential role in the catabolism of branched-chain amino acids (BCAA) (Xu et al., 2023). Notably, previous research found *BCKDHB* expression to be upregulated in breast cancer compared to normal tissue (Zhang and Han, 2017). Additionally, a recent study employing a Cox proportional-hazards model indicated that a higher post-diagnostic intake of dietary BCAAs is associated with increased all-cause mortality risk in CRC patients (Long et al., 2021). However, *BCKDHB* has not yet been included in prognostic classifiers for CRC patient outcomes, nor has its role in oxaliplatin resistance been previously explored.

Another notable gene linked to oxaliplatin resistance and incorporated into one of the prognostic classifiers is *UBTF*. Our results indicate that higher expression of *UBTF* is associated with greater oxaliplatin sensitivity *in vitro* and improved prognosis in Stage II/III CRC patients. Given *UBTF*’s essential role in ribosomal RNA transcription, it’s unsurprising that its knockdown has previously been shown to suppress colon cancer cell proliferation (Tsoi et al., 2017). Additionally, *UBTF* has a known high affinity for cisplatin-DNA adducts, which may act as molecular decoys, redirecting *UBTF* from ribosomal RNA genes and thereby inhibiting their transcription (Hamdane et al., 2015). These findings suggest *UBTF*’s potential importance in CRC progression and drug resistance, although its precise mechanism of action remains uncertain and warrants further investigation.

Interestingly, five genes identified in our correlation analysis were associated with responses to both 5-FU and oxaliplatin. One of these genes showed a positive correlation with the IC50 values of both drugs, while the others exhibited negative correlations with IC50s. Although none of these genes were incorporated into the final gene signatures, they present intriguing targets for further investigation in the context of drug resistance.

The only gene identified to show a positive correlation between its expression and IC50 values for both 5-FU and oxaliplatin was *AVPI1* (arginine vasopressin induced 1). Notably, high levels of *AVPI1* expression were detected in association with cell cycle entry (Kiessling et al., 2010) and its involvement in MAPK pathway activation (Nicod et al., 2002). Although existing research has associated *AVPI1* with the development and progression of cancers like melanoma (Motwani et al., 2021) and prostate cancer (Li et al., 2024), its role in colorectal cancer and resistance to chemotherapy agents such as 5-FU and oxaliplatin has not yet been investigated. On the other hand, a recent study on colorectal cancer cell lines showed that *AVPI1* expression was significantly increased after cisplatin treatment (Saini et al., 2023).

The *DHX33* gene, identified in our correlation analysis, plays a pivotal role in cancer cell proliferation and growth. Regulated by the Wnt/β-catenin pathway, RNA helicase *DHX33* transcriptionally controls key genes involved in the cell cycle, apoptosis, and migration. Previous research has shown that *DHX33* is highly expressed in colon cancer tissues and cell lines, while its deficiency resulted in tumor growth retardation in colon cancer cells in an *in vivo* xenograft model (Wang et al., 2019a; Zhu et al., 2020). Additionally, DHX33 has been found to stimulate Bcl-2 transcription in many human cancer cell lines. At the same time, the acute knockdown of *DHX33* resulted in decreased Bcl-2 protein levels, which ultimately caused mitochondria-mediated cellular apoptosis (Wang et al., 2019b). The fact that cancer cells demonstrate heightened sensitivity to *DHX33* downregulation, highlights its potential as a promising target for cancer therapy (Zhu et al., 2020). However, the connection between DHX33 expression levels and resistance to standard chemotherapy in colorectal cancer has not been thoroughly investigated, introducing a potentially novel perspective in our findings.

Two other genes, *LYRM2* and *WRAP53*, also emerged from our analysis with expression negatively correlated to sensitivity of CRC organoids and cell lines to 5-FU and oxaliplatin. Both are well-established biomarkers of colorectal cancer. *LYRM2* is upregulated in colorectal cancer, where it promotes tumor growth both *in vivo* and *in vitro*. Localized within the inner mitochondrial membrane and matrix, *LYRM2* directly interacts with complex I of the electron transport chain, enhancing its activity and driving oxidative phosphorylation in colorectal cancer cells (Huang et al., 2019).

Similarly, *WRAP53*, a gene encoding a protein critical for DNA repair, is overexpressed in CRC tissues and cell lines (Zhang et al., 2012; Wang et al., 2015). Its knockdown has been shown to suppress tumor cell proliferation and invasion while increasing apoptosis and causing G1 cell cycle arrest (Zhu et al., 2018). The effects of WRAP53 on cancer prognosis are contradictory and may vary depending on the cancer type and treatment approach. For instance, low WRAP53 protein levels have been linked to poor outcomes in breast cancer and are associated with decreased effects of radiotherapy (Egelberg et al., 2023). Conversely, in rectal cancer, high WRAP53 expression in primary tumors and metastases was associated with poor prognosis, except in patients receiving radiotherapy, where it correlated with improved survival (Zhang et al., 2012). Furthermore, while WRAP53 expression is associated with radiotherapy resistance, there is insufficient evidence to establish a direct connection to chemotherapy resistance.

Finally, the *RANGRF* gene, previously linked to Brugada syndrome, represents a novel finding in this context (Campuzano et al., 2014). It has not been previously associated with drug resistance, disease prognosis, or colorectal cancer, making it an intriguing candidate for further investigation.

The third compound examined in this study is SN-38, the active metabolite of irinotecan. It is formed through metabolic conversion primarily facilitated by carboxylesterase 1 and 2 in the liver. SN-38 exerts its anticancer effects by targeting the DNA-topoisomerase I complex, disrupting its catalytic activity, and inducing DNA damage, replication arrest, and subsequent cell death. During its metabolism, SN-38 undergoes glucuronidation by UGT1A1 in the liver, forming the inactive compound SN-38G. Additionally, ABC transporters are critical for the transport and resistance mechanisms of SN-38 (Yu et al., 2005).

Several studies have explored genes associated with CRC resistance to irinotecan and, by extension, SN-38 (Makondi et al., 2017; Kryczka and Boncela, 2022). Unsurprisingly, many of these genes are linked to SN-38 metabolism or its mechanism of action. In this study, we identified several novel genes associated with SN-38 resistance, some of which also have prognostic relevance for CRC patients.

One notable gene is *CD9*, whose expression demonstrated a positive correlation with resistance to SN-38. According to our classifier, higher *CD9* expression in metastatic tissue is associated with poorer overall survival in Stage IV CRC patients. Interestingly, previous studies showed that reduced *CD9* expression in primary tumor tissue was linked to poorer prognosis in CRC (Mori et al., 1998; Kim et al., 2016), while increased *CD9* expression inhibited colon carcinoma cell growth (Ovalle et al., 2007). These contrasting findings highlight the complex role of *CD9* in cancer progression and drug resistance, suggesting its effects may vary based on tumor stage, tissue context, or metastatic status. Further research is warranted to clarify *CD9*’s function in SN-38 response and its potential as a therapeutic target in CRC.

Another noteworthy gene is *TAS2R4*, encoding Taste 2 Receptor Member 4, involved in various Taste Receptor Pathways, defined in our pathways analysis. The potential role of bitter signaling in cancer progression and drug resistance is an emerging area of research. Bitter taste receptors, including *TAS2R4*, have been implicated in the progression of various cancer types (Costa et al., 2023). It is worth noting that the activation of bitter taste receptors in cancer cells is predominantly associated with anti-cancer effects, and decreased expression of some TAS2Rs, such as TAS2R4, 5, 9, 10 or 14, is associated with poor prognosis (Zehentner et al., 2021), while increased expression counteracts carcinogenesis (Seo et al., 2017). Notably, bitter taste receptors may regulate ABC transporters, proteins that play a known role in irinotecan and SN-38 resistance (Yu et al., 2005). Moreover, bitter taste compounds can be either substrates or inhibitors of ABC transporters and thus play the role of reliable targets for reversing chemoresistance in cells of different types of cancer (Huang et al., 2014; Stern et al., 2018; Ravisankar et al., 2019). Our analysis revealed that elevated *TAS2R4* expression was associated with reduced resistance to SN-38 and a more favorable prognosis in CRC patients. While no studies directly link *TAS2R4* to CRC, its involvement in breast cancer progression has been discussed (Singh et al., 2014; 2020). Our findings underscore the importance of further studies to elucidate the function of bitter taste receptors, particularly *TAS2R4*, in CRC and its response to chemotherapy.

Studying the role of individual genes in metastasis and therapy resistance is crucial for advancing our understanding of the molecular mechanisms cancer cells use to evade treatment. These insights can also help identify novel therapeutic targets. However, single genes rarely offer sufficient predictive accuracy for determining patient outcomes. In contrast, multi-gene expression signatures provide a more comprehensive and reliable approach to predict individual outcomes.

Current research in this field predominantly focuses on stages II and III colorectal cancer. Adjuvant chemotherapy is a standard treatment for stage III CRC, yet its absolute benefit is limited, with only an estimated 30% of patients experiencing a meaningful survival advantage (Taieb and Gallois, 2020). For stage II CRC, the role of adjuvant chemotherapy remains controversial due to its minimal survival improvement, combined with significant side effects and substantial financial costs (Chan and Chee, 2019). These challenges underscore the urgent need for precise biomarkers to optimize treatment strategies.

The effort to predict CRC outcomes using gene expression signatures began in the era of microarray technology (Wang et al., 2004; Barrier et al., 2006; Smith et al., 2010). Despite significant progress, recent studies continue to highlight the importance and relevance of this approach (Oki et al., 2021; Ren et al., 2022; Peixoto et al., 2023; Rokavec et al., 2023). Most classifiers developed to date focus on genes associated with the metastatic potential of cancer cells and relapse risk. However, successful clinical implementation of gene expression-based classifiers will require extensive validation and the creation of more accurate and robust signatures (Ahluwalia et al., 2021). These tools should aim to refine therapy selection by distinguishing responders from non-responders and facilitating personalized treatment plans. As these methodologies are refined, they hold the potential to revolutionize CRC management by improving patient outcomes and minimizing unnecessary treatment burdens.

In this study, we developed a gene expression signature based on the expression of genes linked to sensitivity to key chemotherapeutic agents used in colorectal cancer treatment, identified through *in vitro* experiments. This signature demonstrated a robust ability to differentiate between Stage II/III CRC patients treated with adjuvant chemotherapy who had favorable versus unfavorable prognoses. Remarkably, in multivariate analysis, the predicted status derived from this signature emerged as the only significant factor influencing relapse risk. Our proposed 12-gene classifier was directly compared to TNM staging and the CIT classification (Marisa et al., 2013) in a multivariate analysis, where it demonstrated superior prognostic accuracy in the cohort of treated Stage II/III CRC patients. Notably, it emerged as the only significant prognostic factor, with HR of 7.12 (95% CI: 3.73–13.6). Furthermore, the HR values observed for our classifier were markedly higher than those reported for another widely used molecular classification system, CMS (Guinney et al., 2015). This indirect comparison suggests that our classifier may offer enhanced utility for assessing recurrence risk specifically within the population of treated CRC patients.

In contrast, the same gene expression signature, as expected, showed poor predictive performance in a cohort of untreated Stage II/III CRC patients. In this group, the stage of the disease—a well-established risk factor—was the sole significant predictor of outcomes. These results underscore that the prognosis of treated Stage II/III CRC patients is primarily determined by their response to therapy. This study highlights the importance of integrating genes associated with treatment response, in addition to those related to metastatic potential, into gene expression signatures. Such an approach could significantly enhance the precision of existing prognostic tools and provide more actionable insights for guiding adjuvant therapy decisions, paving the way for more personalized and effective treatment strategies in Stage II/III colorectal cancer.

Compared to the extensive efforts dedicated to developing gene expression signatures to predict outcomes for Stage II/III colorectal cancer patients, relatively few studies have focused on similar classifiers for Stage IV CRC patients (Wada et al., 2022). One possible explanation is the established and substantial benefit of chemotherapy in metastatic CRC, making it standard practice for all patients. However, despite this, treatment outcomes for metastatic CRC remain suboptimal, highlighting the urgent need for more personalized therapeutic approaches.

In this study, the gene expression signature developed for metastatic CRC patients demonstrated excellent performance, reinforcing the idea that chemotherapy response is a critical determinant of treatment success in Stage IV CRC. Notably, patients classified as having a favorable prognosis exhibited remarkably high survival rates for metastatic CRC. Our analysis suggests that only about one-third of metastatic CRC patients derive significant benefit from chemotherapy. This finding raises the critical question of how to effectively treat the remaining two-thirds who show an unfavorable response to standard therapy.

For these patients, alternative treatments such as targeted therapies or immunotherapies may be necessary. The development of specific biomarkers to guide these therapies is an area of active research (Shiravand et al., 2022; Mustafa et al., 2024b). For targeted therapy, particularly involving off-label drugs (Liu et al., 2024), next-generation sequencing (NGS) (Jan et al., 2022) and *in vitro* functional assays using tumor organoids have shown promise (Ooft et al., 2021; Jensen et al., 2023; Martini et al., 2023).

Similarly, for immunotherapy, various strategies are emerging. These include assays based on gene expression profiling, circulating tumor DNA analysis, and other innovative approaches (Hou et al., 2022). The integration of such tools with gene expression signatures could enable more precise and effective therapeutic decisions, transforming the management of Stage IV CRC by tailoring treatment to individual patient profiles.

Furthermore, additional analyses were conducted to elucidate the biological mechanisms underlying our prognostic classifiers. Our findings reveal distinct stage-specific mechanisms driving prognostic differences in CRC. In Stages II-III, tumors with an unfavorable prognosis were enriched in pathways related to oxidative phosphorylation, thermogenesis, and extracellular matrix (ECM) organization, reflecting increased metabolic activity and structural remodeling that support tumor progression (Mao et al., 2018). In contrast, Stage IV tumors demonstrated enrichment in immune-related and stress-response pathways, indicative of the adaptive and inflammatory characteristics of metastatic cancer (Burgos-Molina et al., 2024).

Immune and stromal scores effectively distinguished prognosis groups at early stages, with favorable cases exhibiting lower scores and higher tumor purity. This suggests a less complex and less immunologically active tumor microenvironment in favorable prognosis tumors. However, in advanced CRC, these scores showed no significant differences between prognosis groups, indicating a stable, immune-enriched microenvironment irrespective of prognosis.

These findings underscore the dynamic influence of the tumor microenvironment across CRC stages. While immune and stromal components play a pivotal role in shaping prognosis at early stages, their impact diminishes as tumors adapt and progress. This highlights the necessity for stage-specific therapeutic approaches to effectively target the evolving biological and immune landscapes of CRC (Huijbers et al., 2013).

## 5 Conclusion

This study highlights the potential of integrating gene expression signatures based on *in vitro* drug sensitivity data into the clinical management of colorectal cancer. By focusing on genes linked to chemotherapeutic response, our classifiers effectively distinguished favorable and unfavorable prognoses in both Stage II/III and Stage IV CRC patients. Notably, chemotherapy response emerged as a major determinant of survival, underscoring the value of treatment-response-based signatures. In Stage II/III CRC, these classifiers could guide adjuvant therapy decisions by identifying patients who are most likely to benefit from treatment, thereby optimizing therapeutic strategies and minimizing unnecessary toxicity. For metastatic CRC the classifiers can help identify non-responders and underscore the need for alternative therapies, such as targeted or immunotherapy. While our results demonstrate that *in vitro* sensitivity data can inform clinical decisions, reproducibility remains a challenge. Combining data from various models, such as organoids and cell lines, is essential to enhance robustness and predictive accuracy. Continued refinement and validation of these approaches will pave the way for more personalized and effective treatments of CRC.

## Data Availability

The datasets presented in this study can be found in online repositories. The names of the repository/repositories and accession number(s) can be found in the article/Supplementary Material.
