# Supplementary material for: Predicting patient outcomes with gene-expression biomarkers from colorectal cancer organoids and cell lines
Source: Front Mol Biosci. 2025 Jan 15;12:1531175. doi: 10.3389/fmolb.2025.1531175 (PMC11774744; doi:10.3389/fmolb.2025.1531175)
Supplement: Supplementary file 1 [file DataSheet1.docx]

**
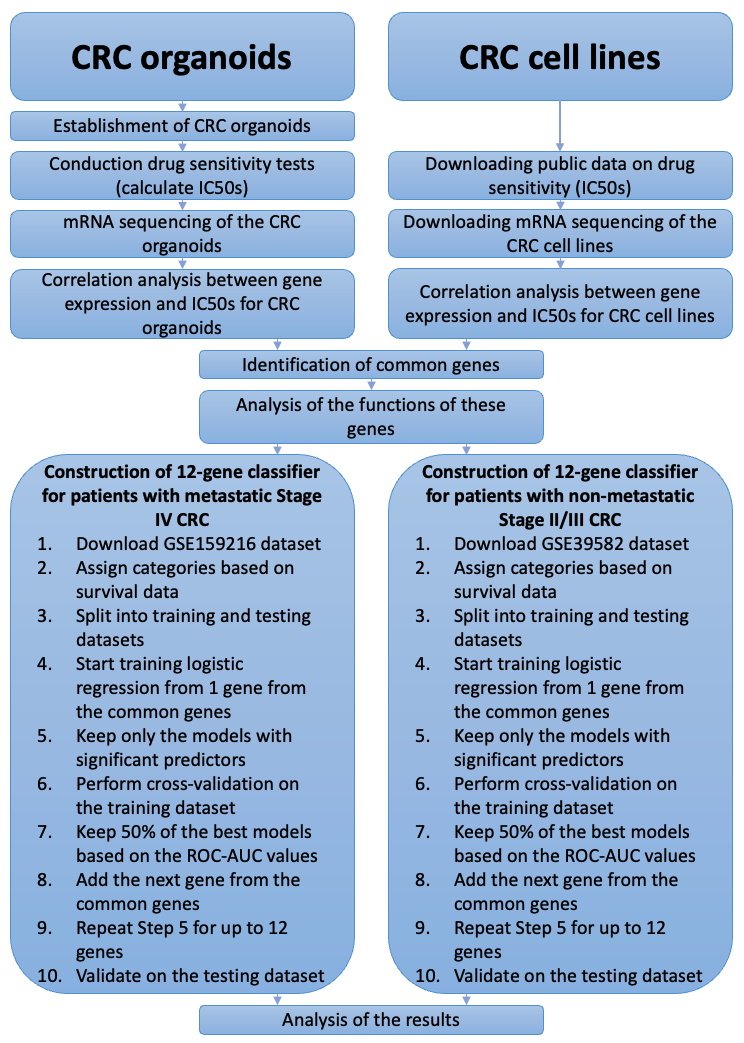
**

**Figure S1.** General flowchart of the study.

**Table S1.** Clinical parameters of the patients included in the study.

| **Patient** | **Age** | **Sex** | **Localization of the metastatic lesion** | **Previous treatment** |
| --- | --- | --- | --- | --- |
| P1 | 66 | Female | Liver | Capecitabine, 5-FU, Oxaliplatin, Irinotecan, Bevacizumab |
| P2 | 54 | Female | Lung | Capecitabine, 5-FU, Oxaliplatin, Bevacizumab |
| P3 | 45 | Female | Lung | Capecitabine, 5-FU, Oxaliplatin, Irinotecan |
| P4 | 47 | Male | Liver | Capecitabine, 5-FU, Oxaliplatin, Irinotecan, Bevacizumab |
| P5 | 37 | Male | Liver | 5-FU, Oxaliplatin, Irinotecan, Cetuximab, Bevacizumab, Regorafenib |
| P6 | 55 | Male | Liver | 5-FU, Oxaliplatin, Irinotecan |


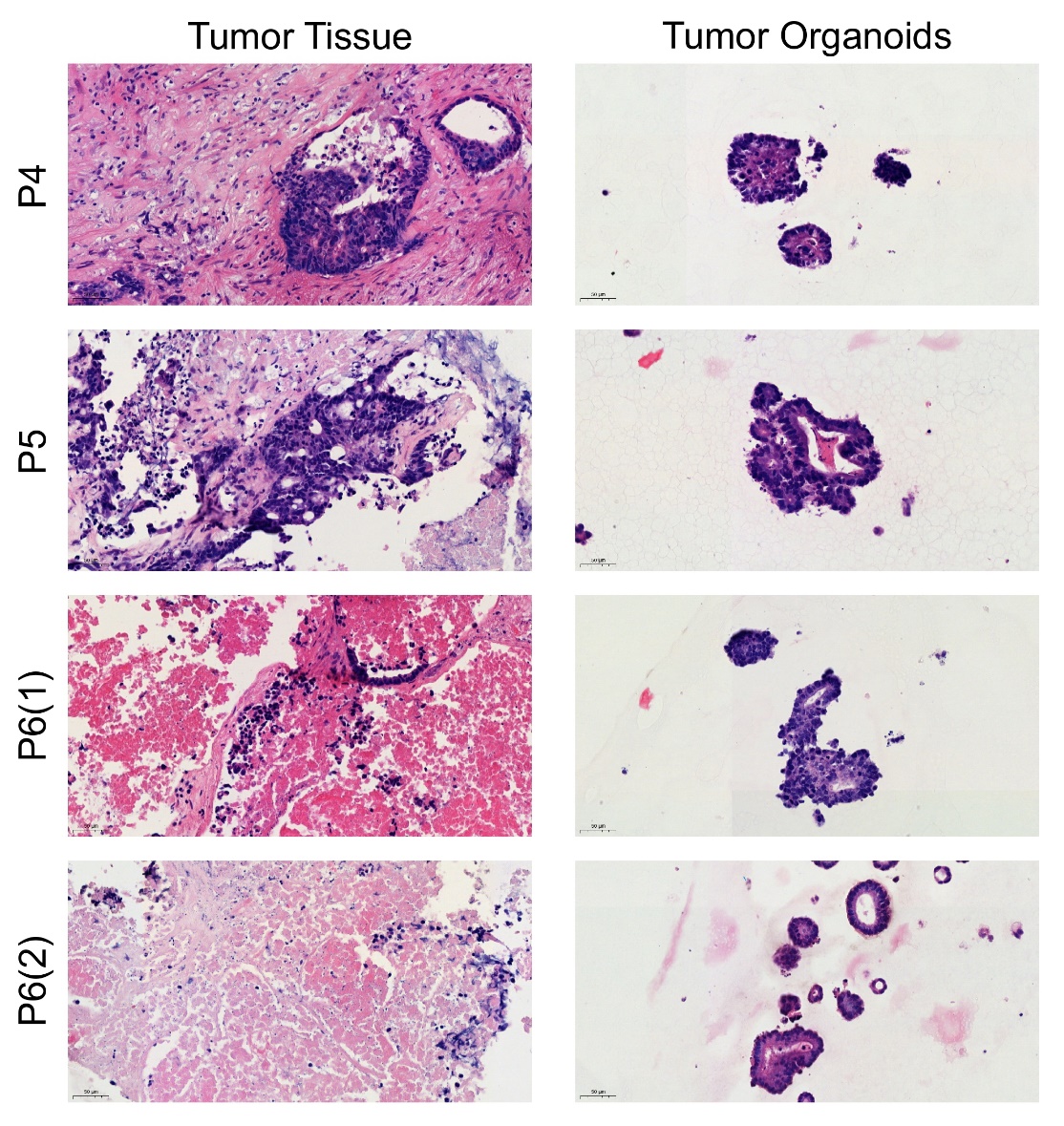


**Figure S2.** Histological analysis of H&E-stained slides from the initial tissue and organoids. P4–P6 refer to patient numbers. Scale bars represent 50 μm.

**
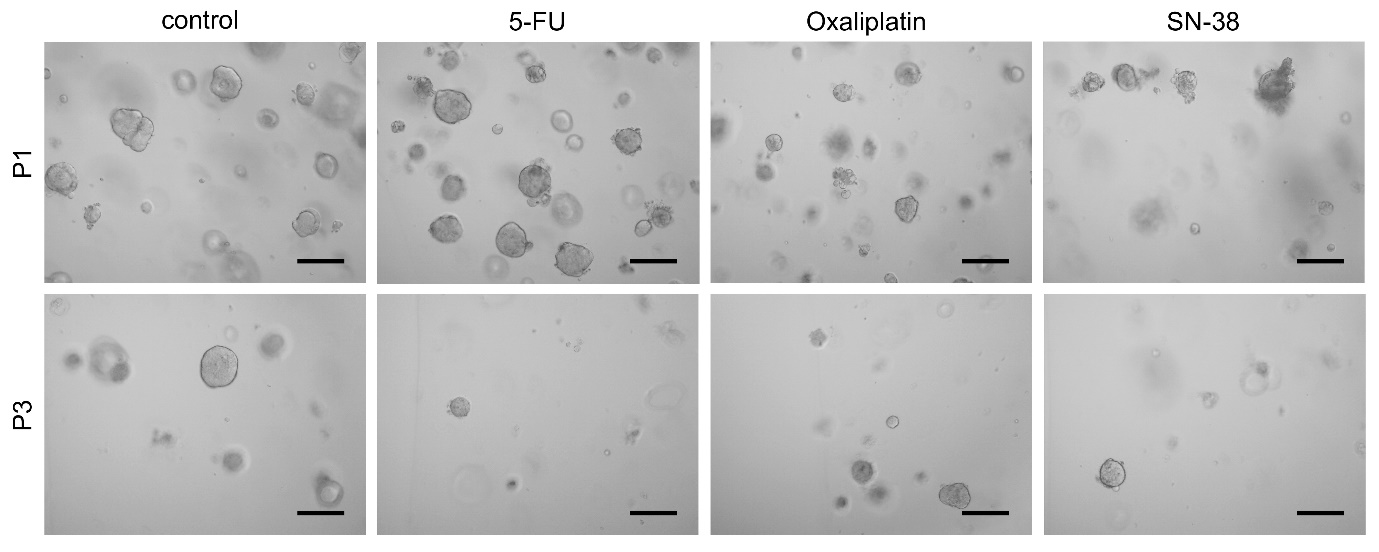
**

**Figure S3.** Brightfield images of the established CRC PDOs after treatment with 5-FU (150 μM), Oxaliplatin (50 μM) or SN-38 (50 μM) for 72 hours. P1 and P3 refer to patient numbers. Scale bars represent 200 μm.


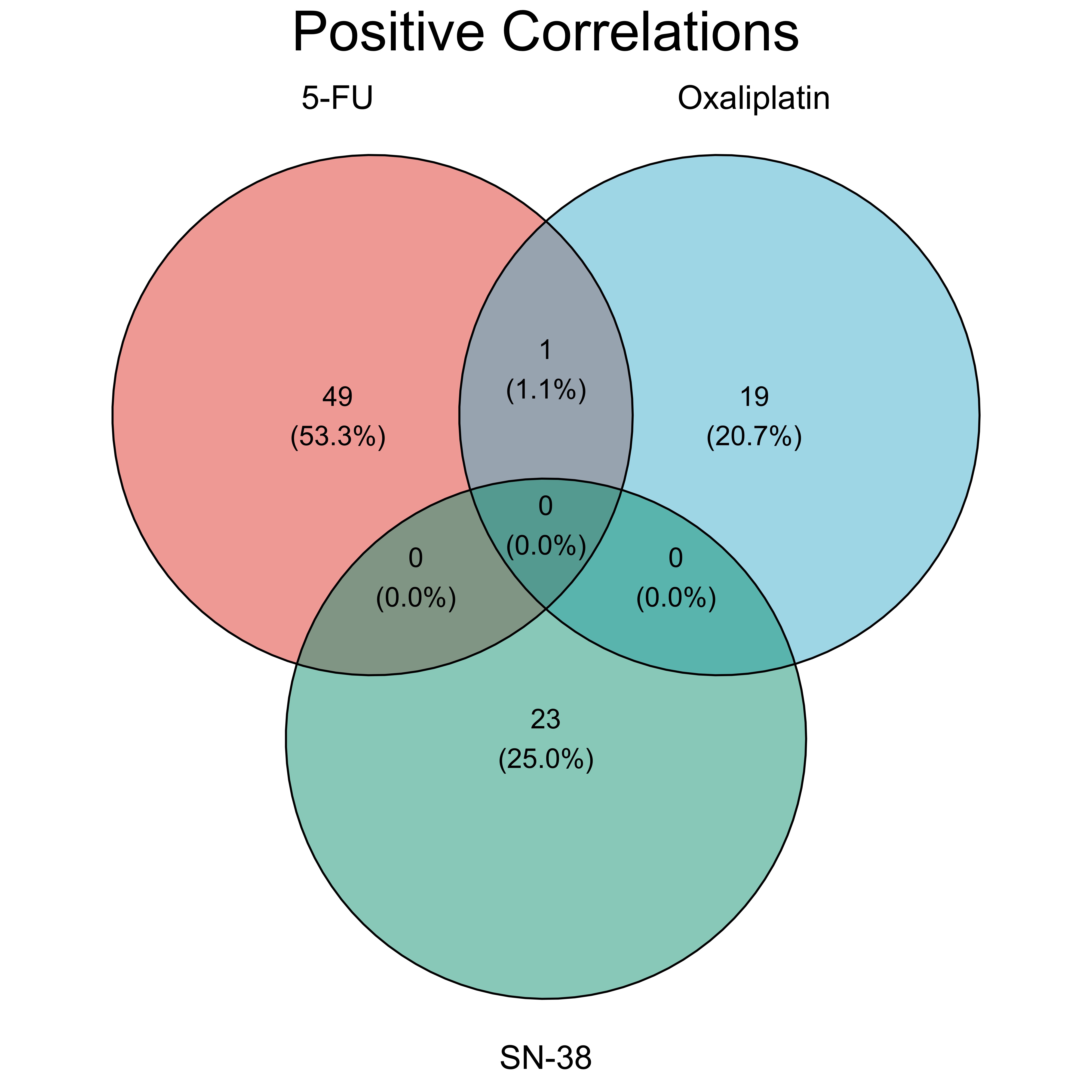

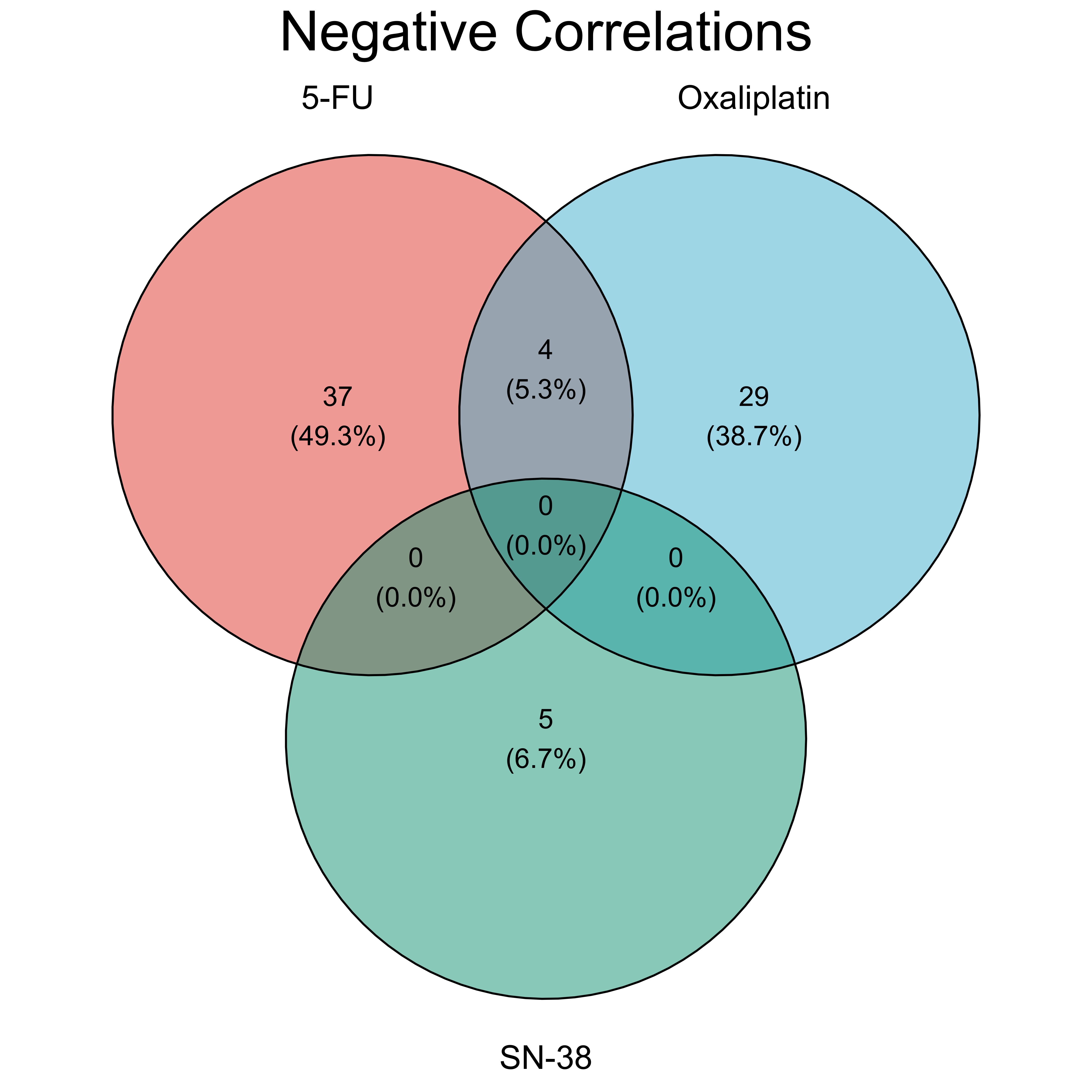


**Figure S4.** Venn diagrams showing the overlap of significantly correlated genes in a single direction (either positive or negative) across different standard-of-care (SOC) drugs.

| 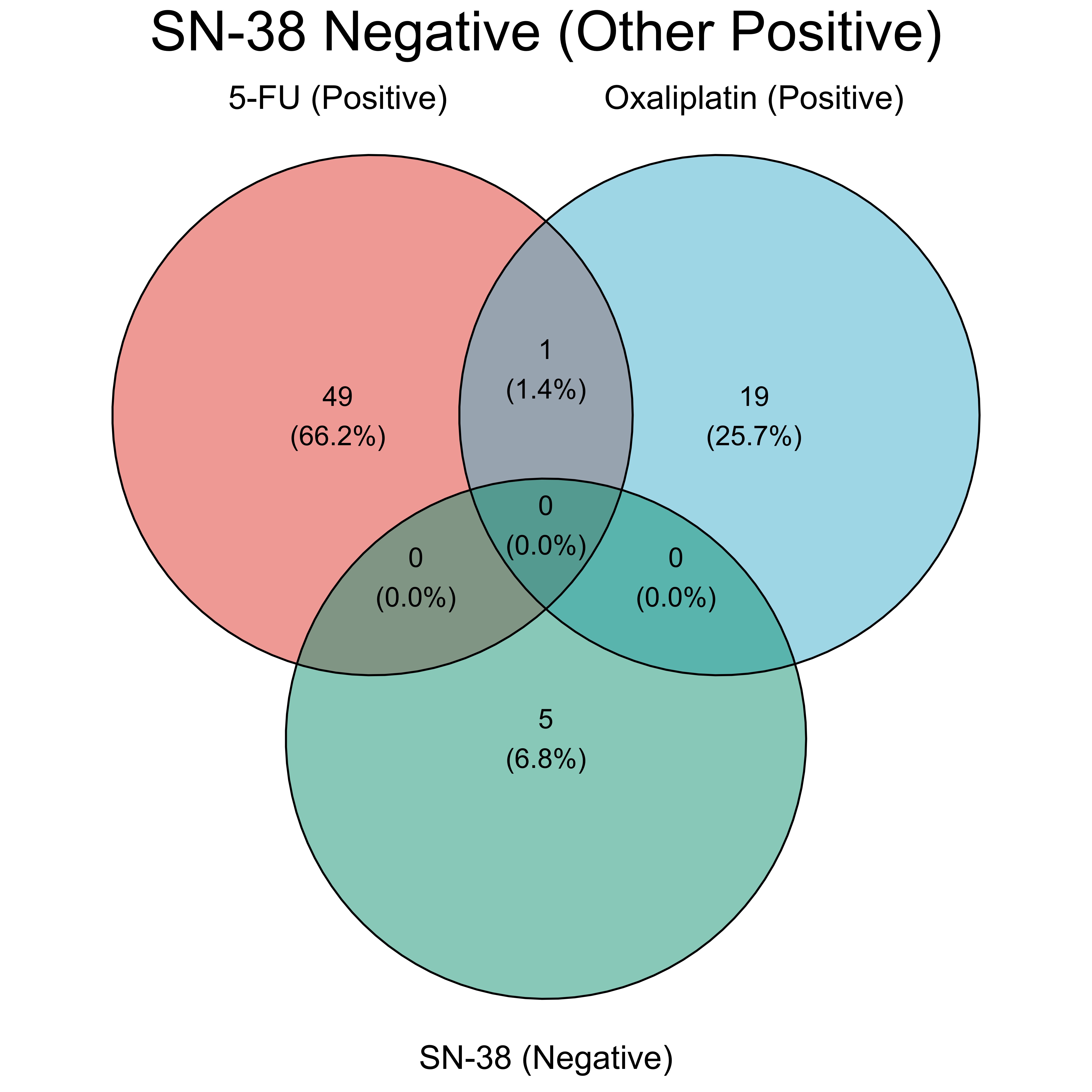 | 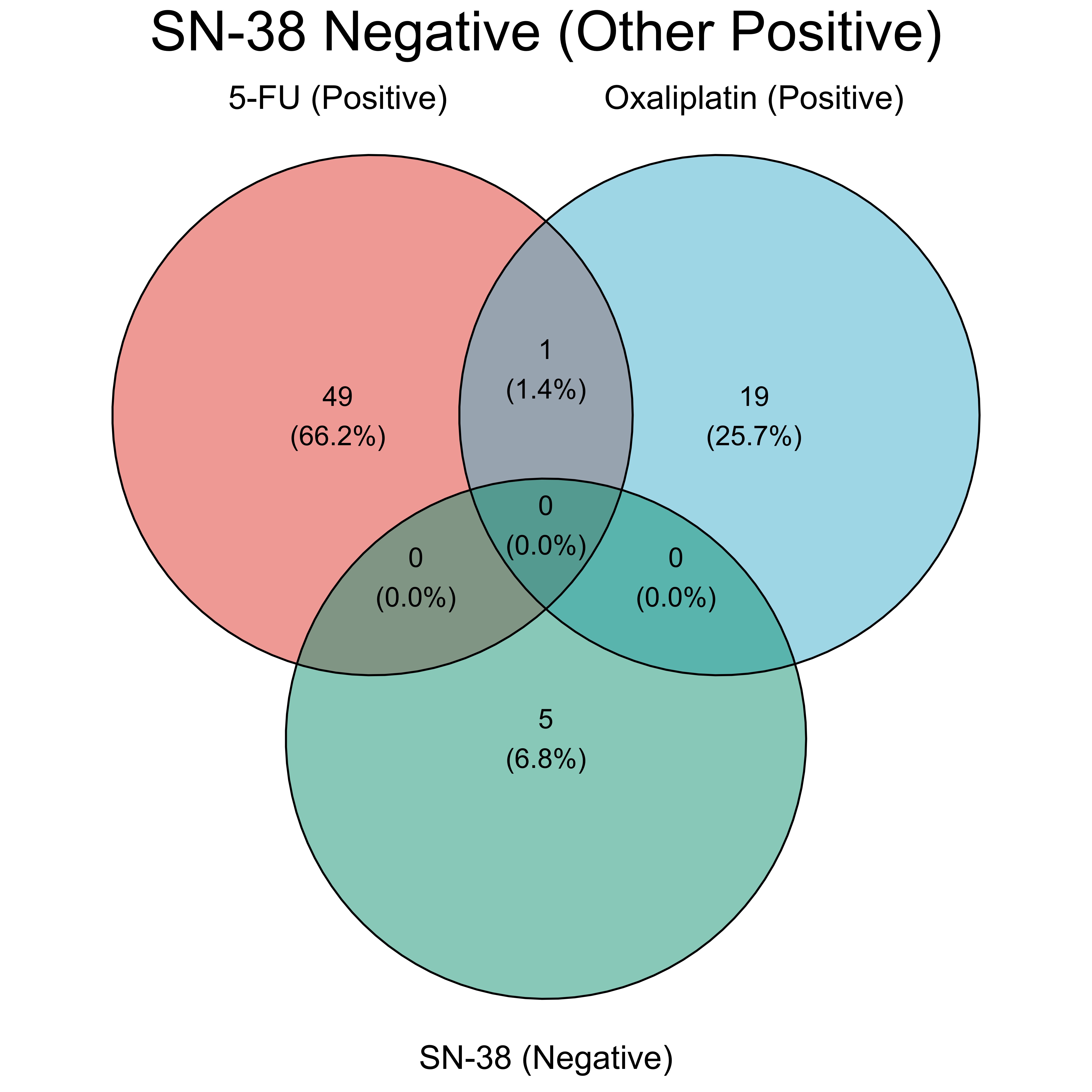 |
| --- | --- |
| 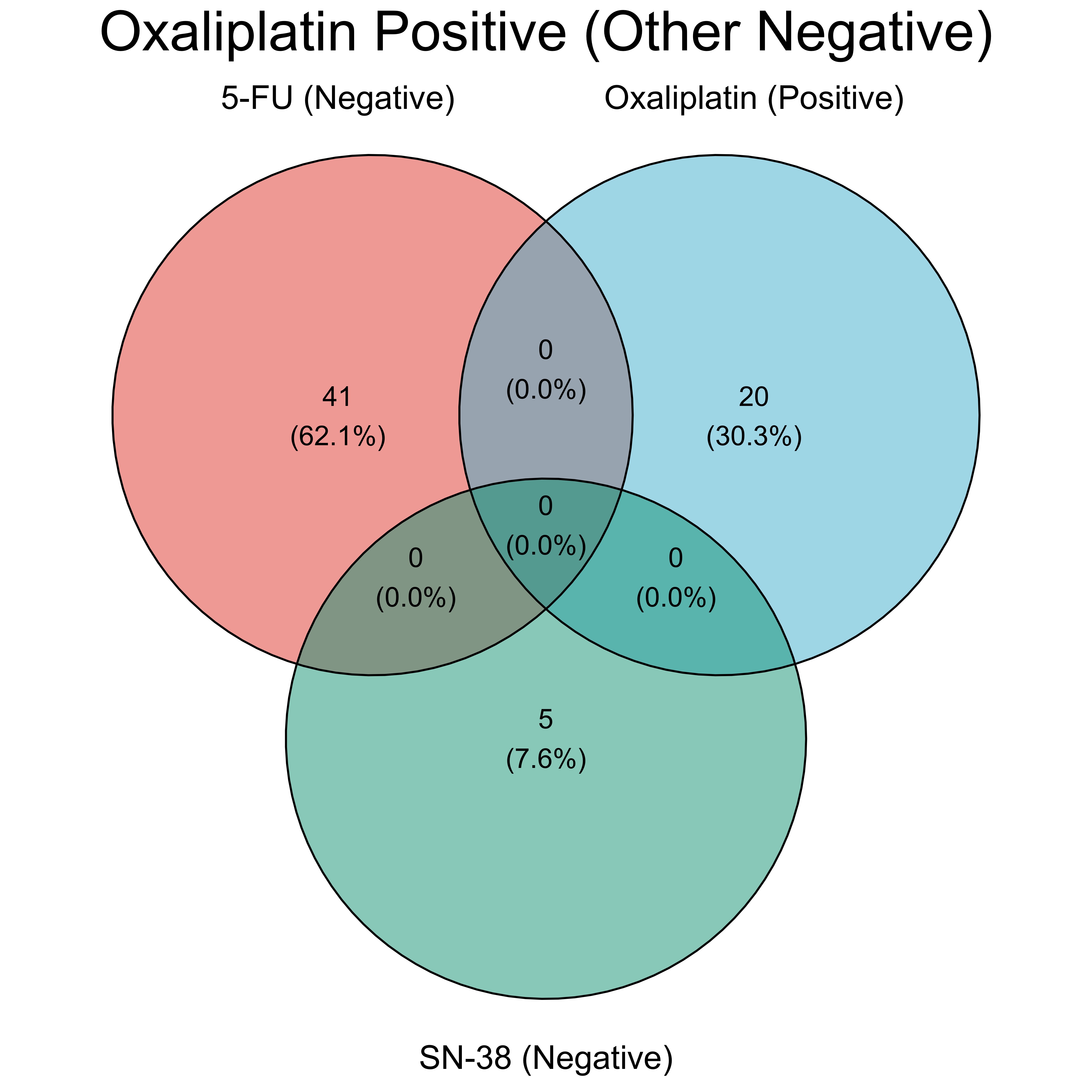 | 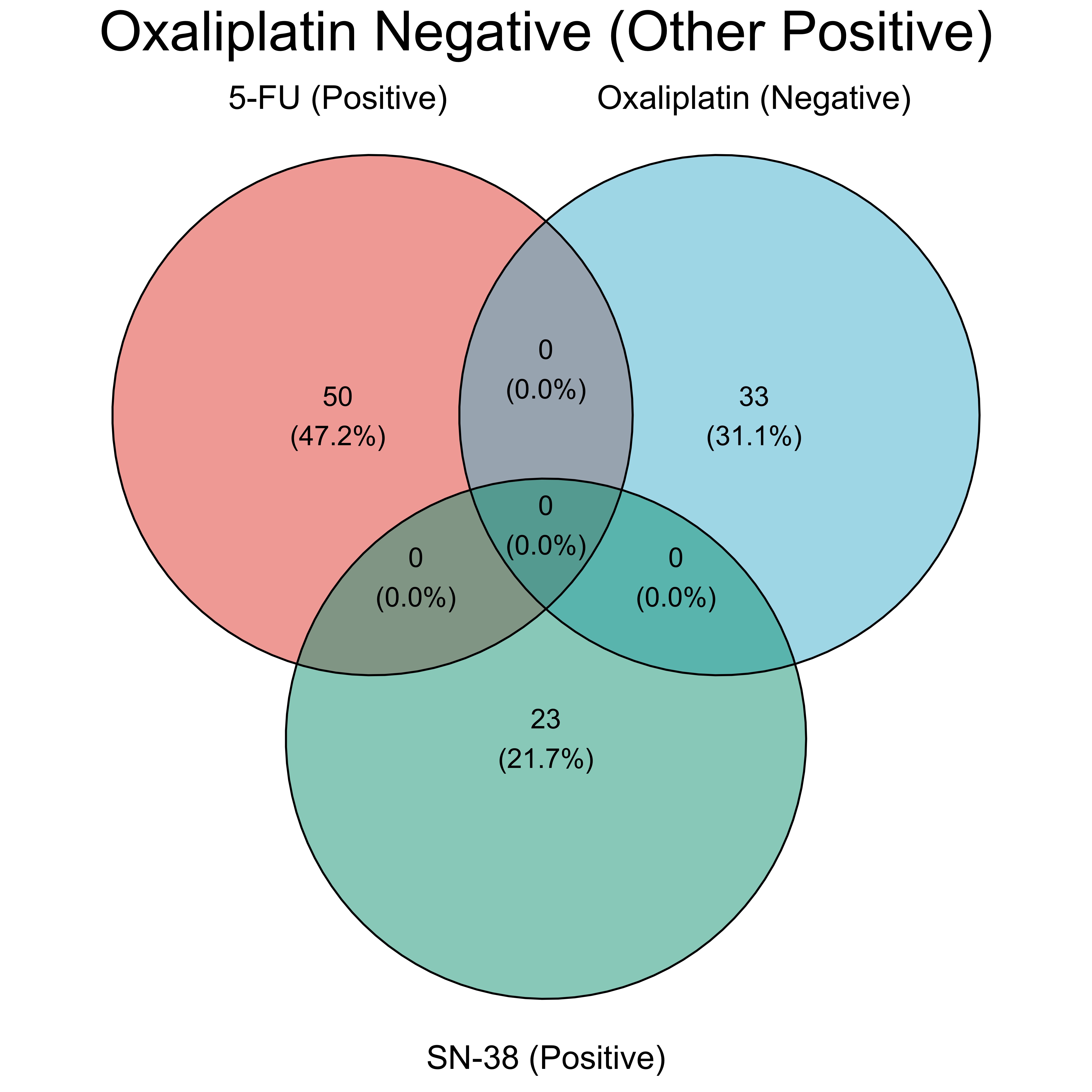 |
| 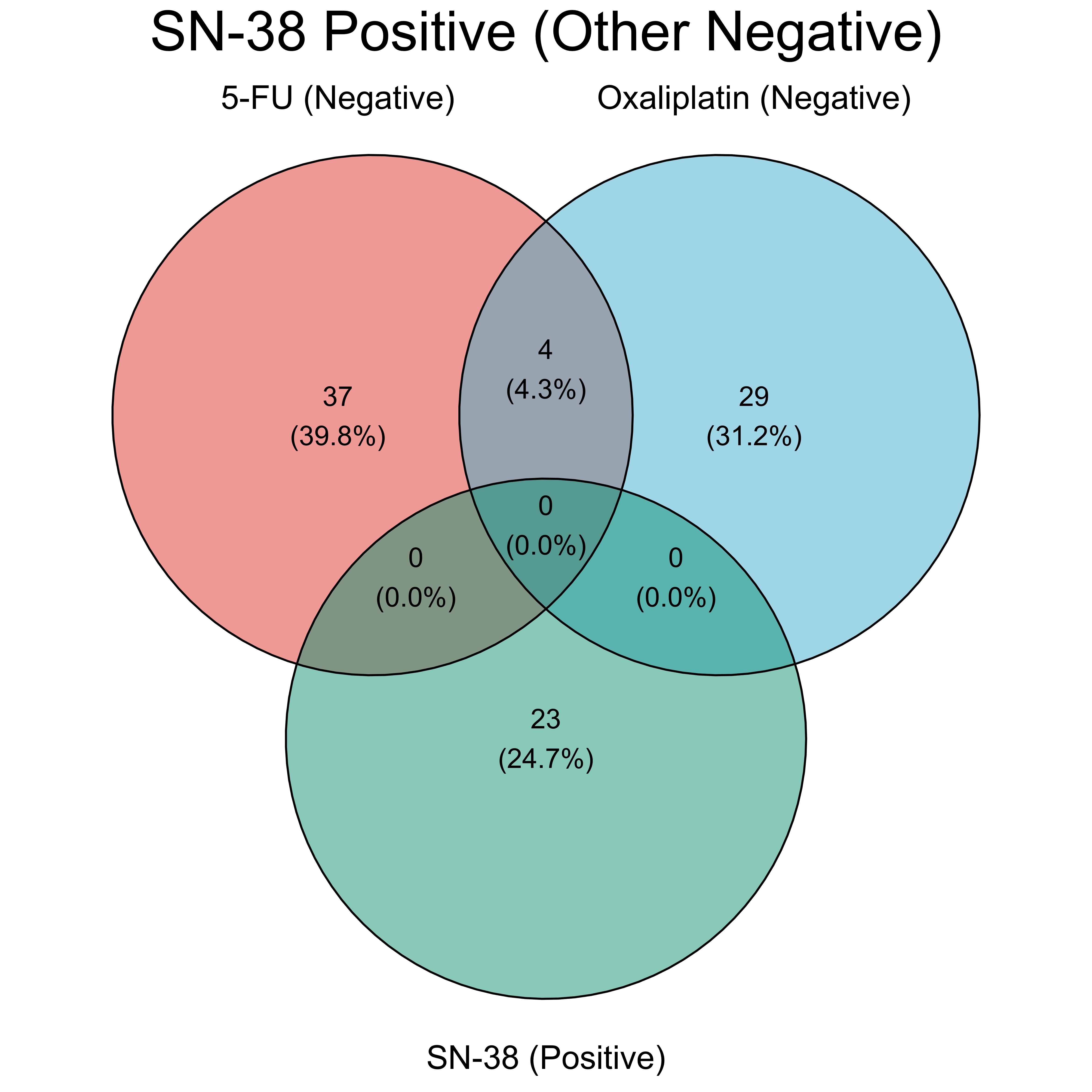 | 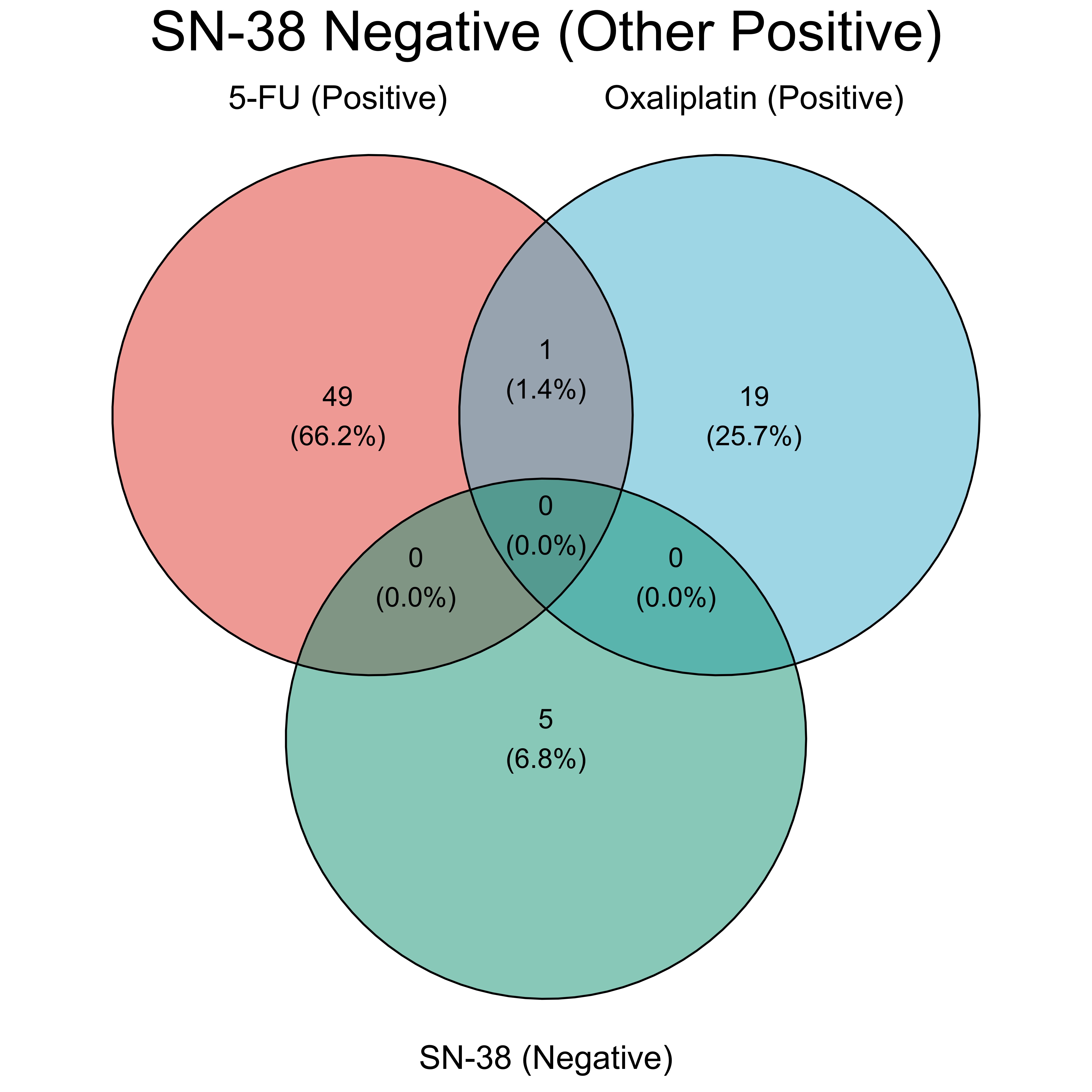 |

**Figure S5.** Venn diagrams showing the overlap of genes that are significantly correlated in one direction for a specific standard-of-care (SOC) drug, but in the opposite direction for other SOC drugs.

**
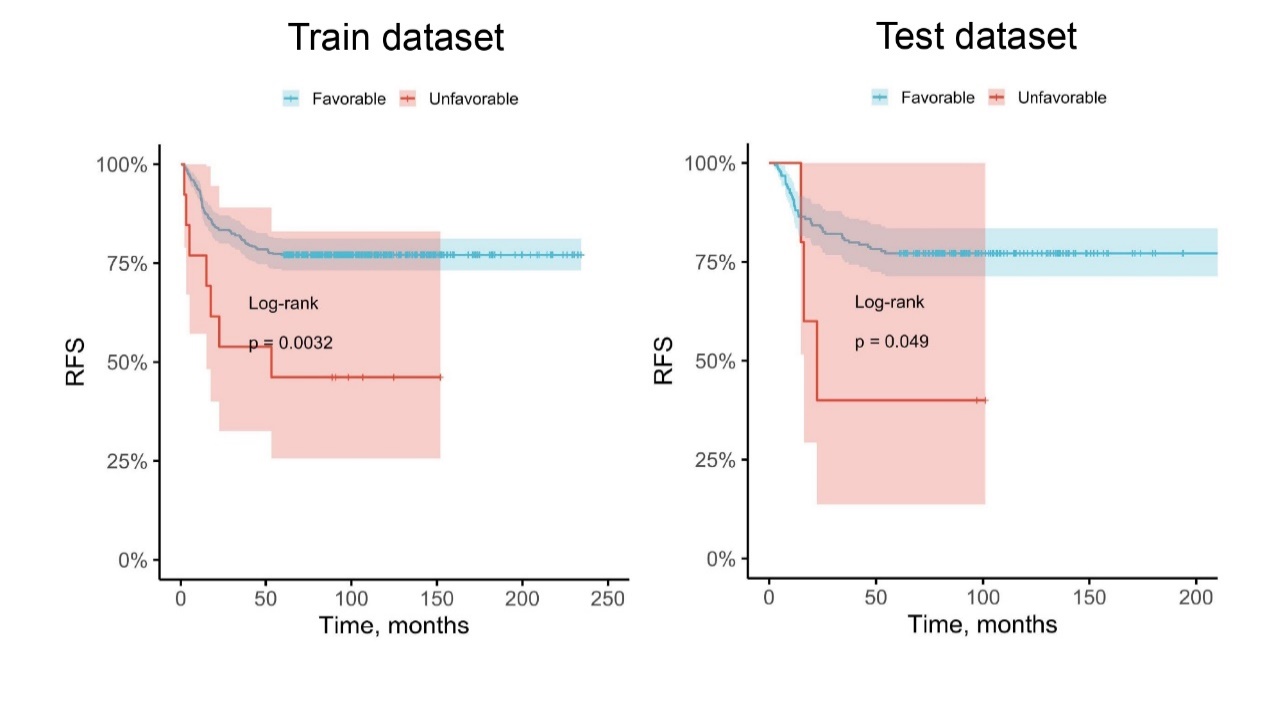
**

**Figure S6.** Kaplan-Meier plots for the training (n = 441) and test (n = 189) datasets for additional validation of classifier for Stage II/III CRC patients using mRNA-seq (E-MTAB-12862 dataset).

|  | 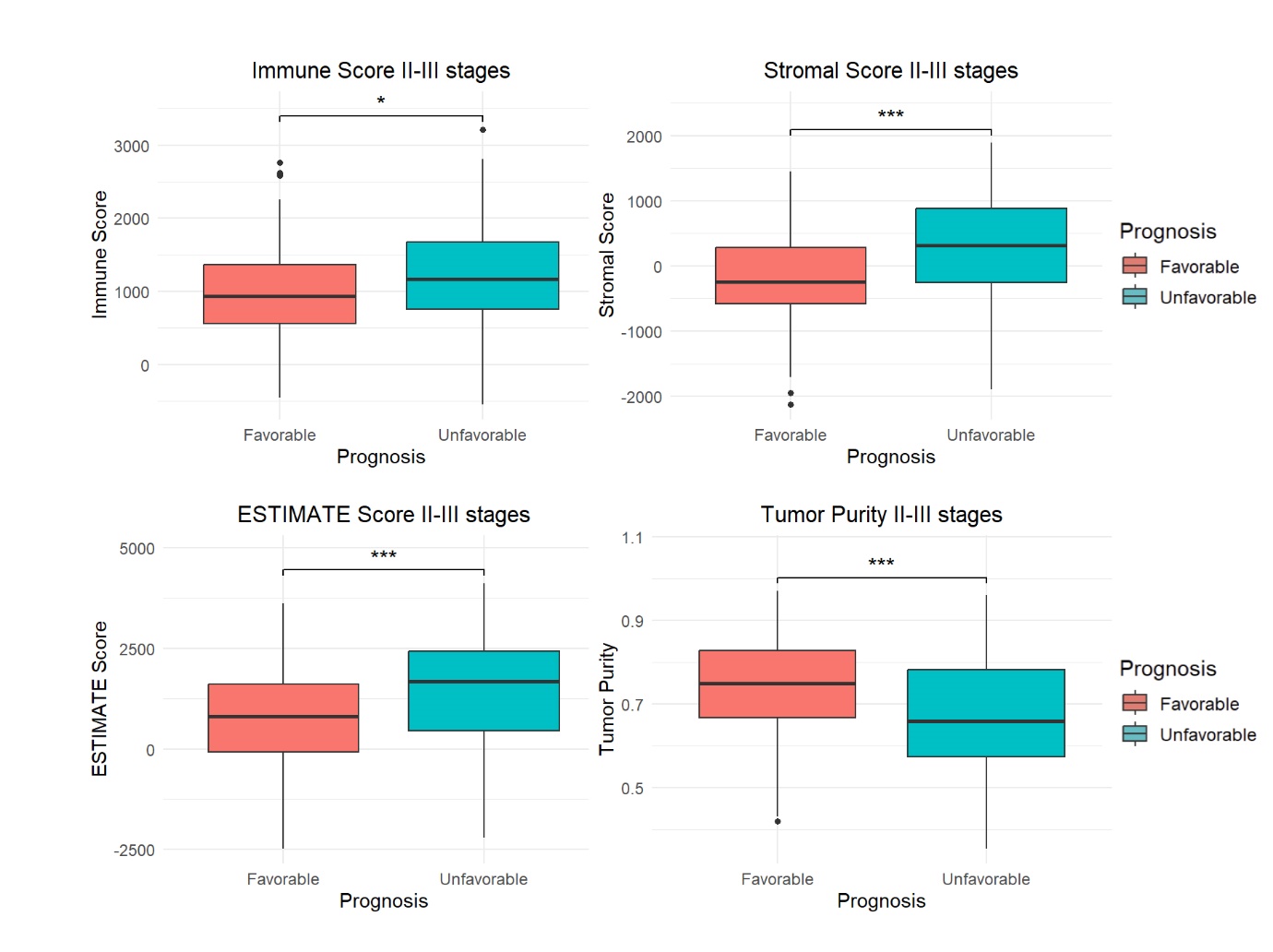 |
| --- | --- |

**Figure S7.** Scores calculated by ESTIMATE for Stage II/III CRC patients, stratified by prognosis. Statistical significance was calculated by t-test, and the following significance levels are indicated in the plots: p < 0.05 (*), p < 0.01 (**), and p < 0.001 (***).

|  | 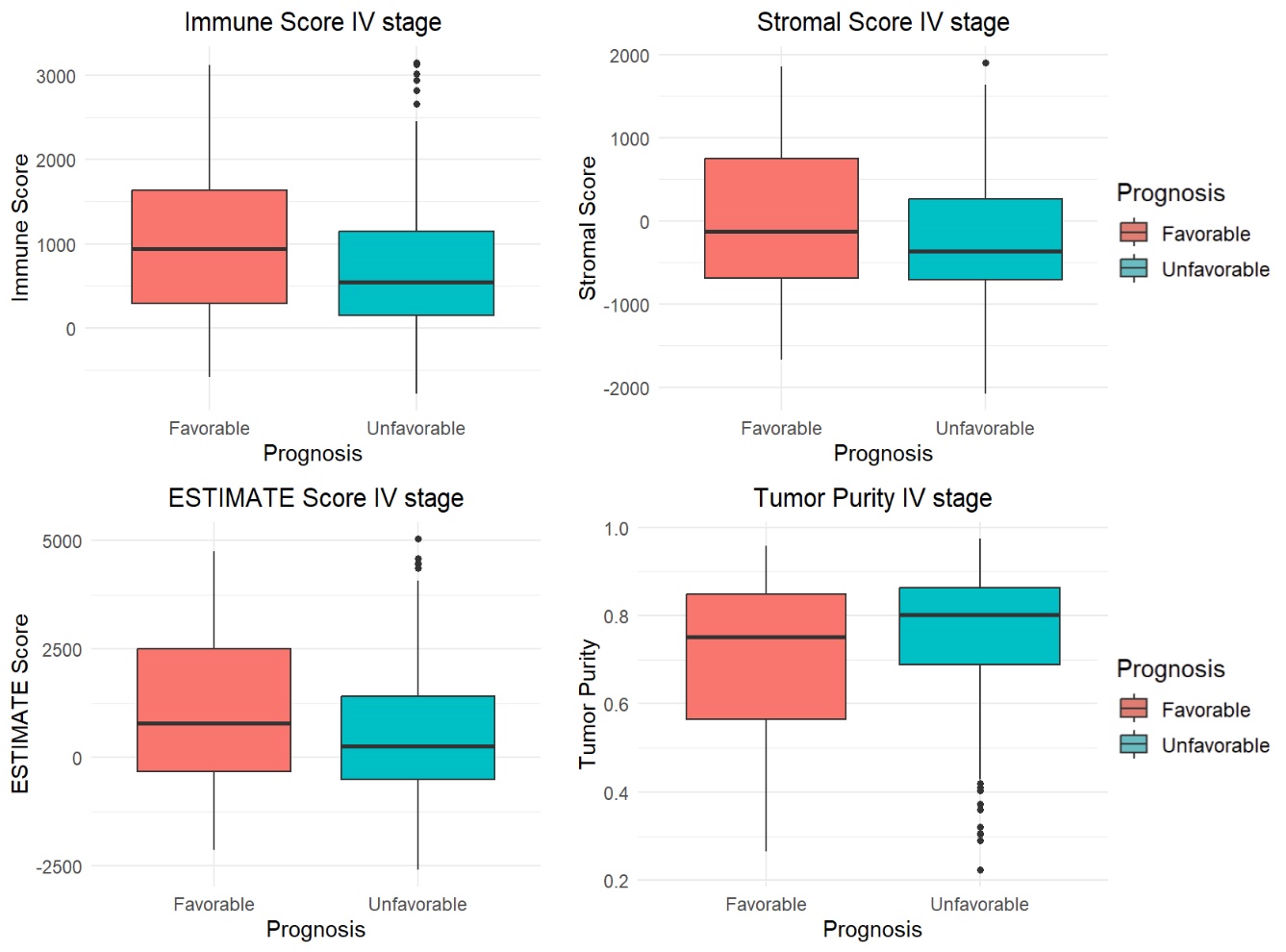 |
| --- | --- |

**Figure S8.** Scores calculated by ESTIMATE for Stage IV CRC patients, stratified by prognosis.

|  | 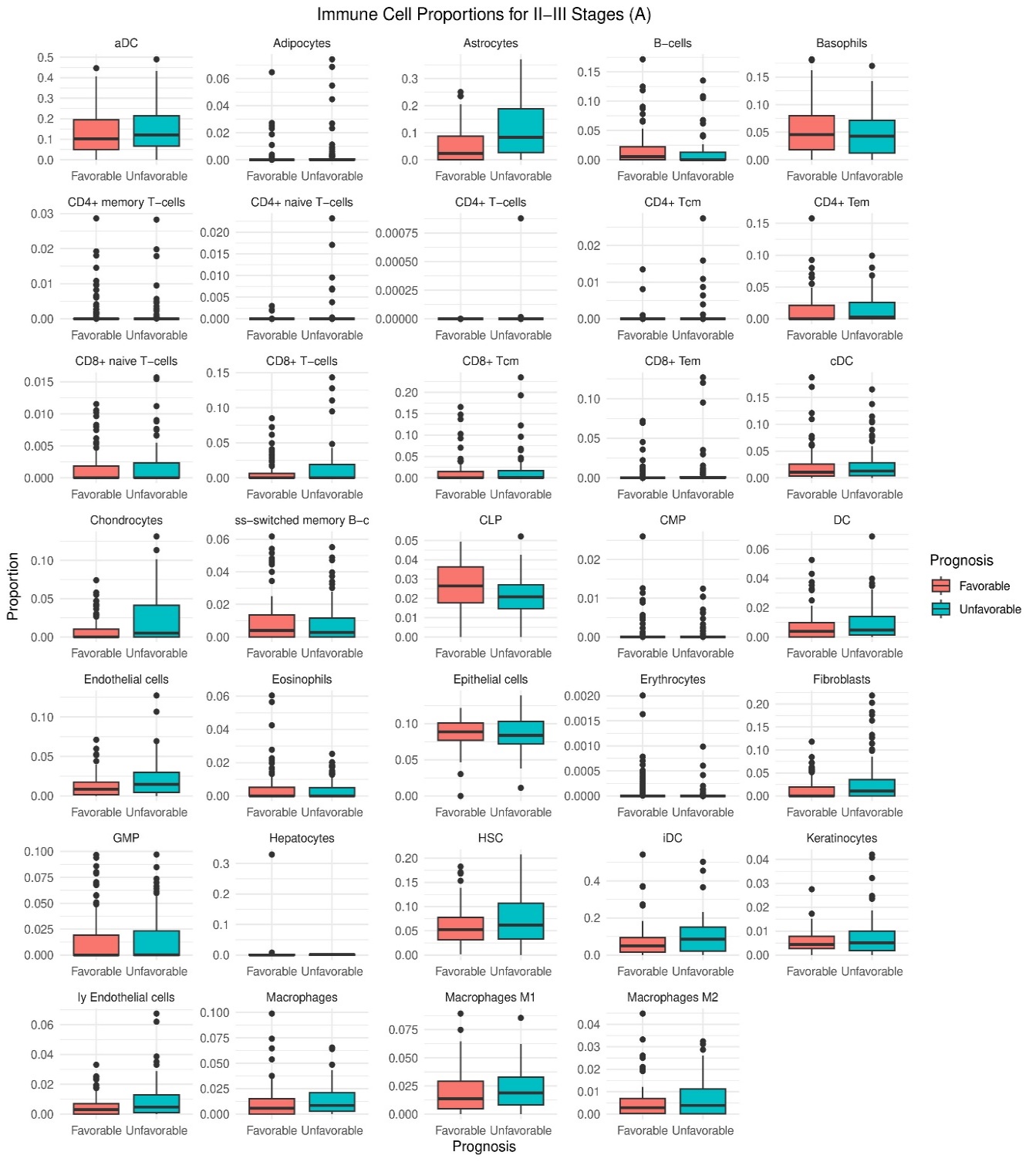 |
| --- | --- |

**Figure S9A.** Boxplots illustrating the proportions of various immune and stromal cell types across samples with favorable and unfavorable prognosis for Stage II/III CRC patients.

|  | 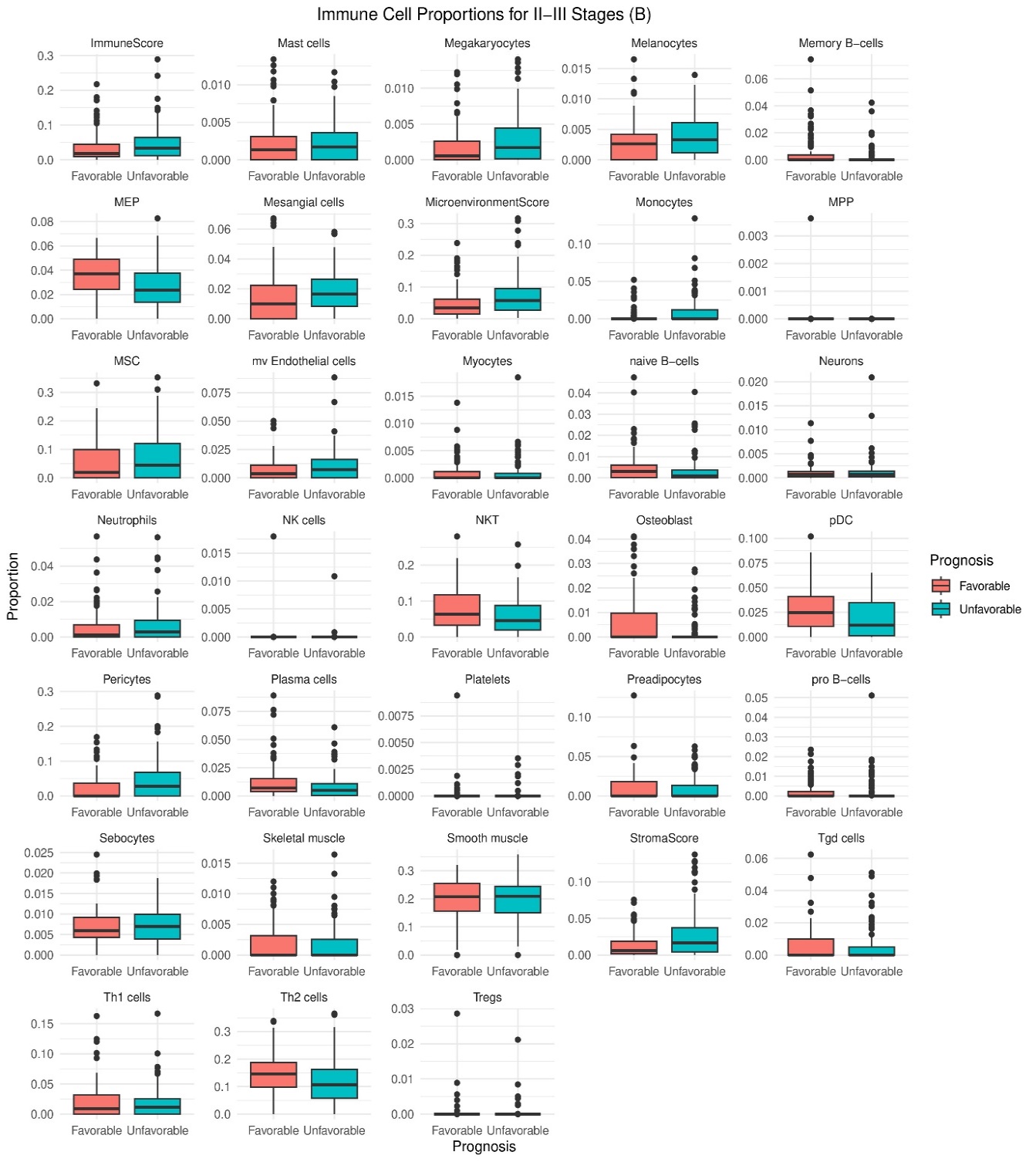 |
| --- | --- |

**Figure S9B.** Boxplots illustrating the proportions of various immune and stromal cell types across samples with favorable and unfavorable prognosis for Stage II/III CRC patients.

|  | 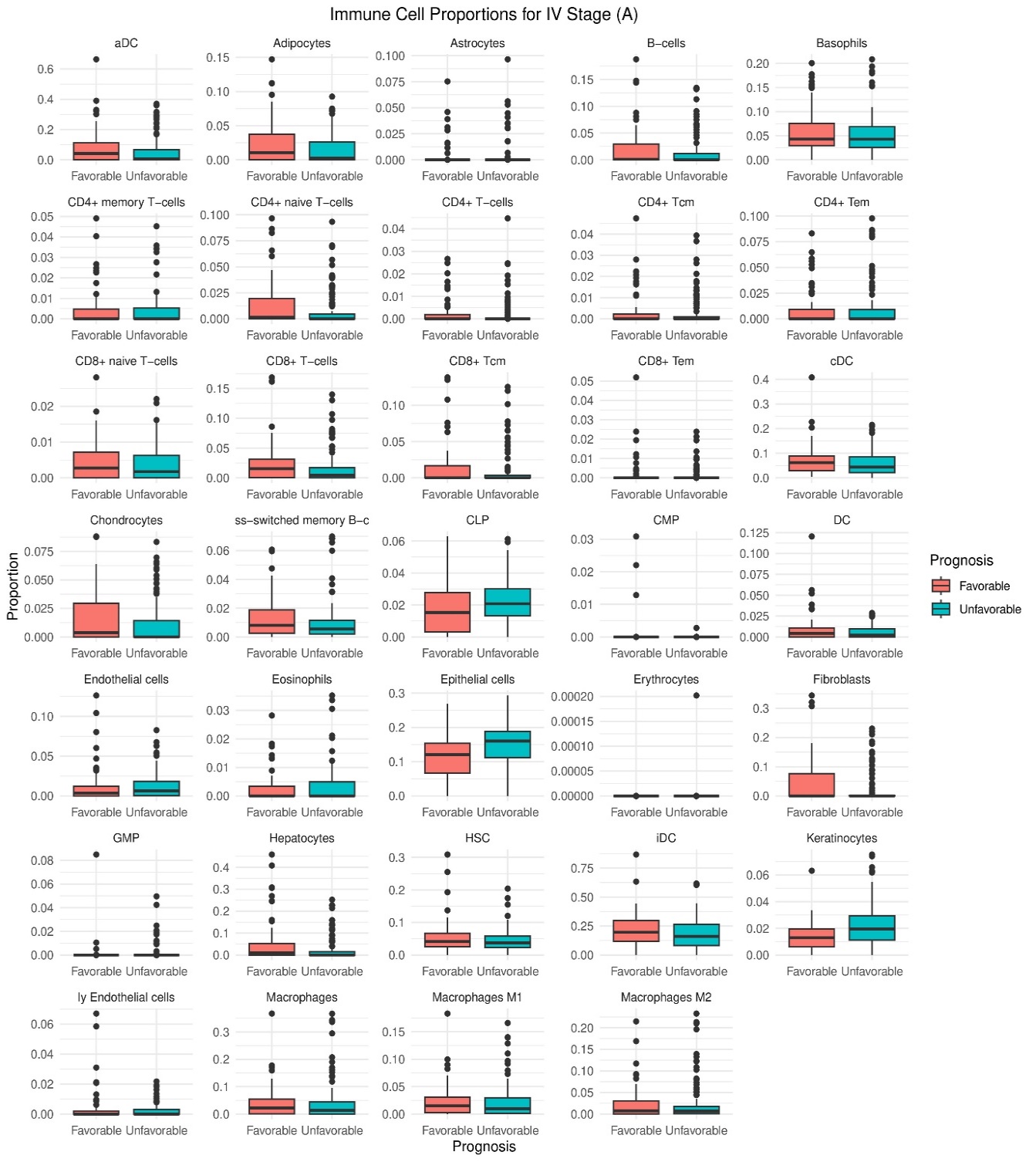 |
| --- | --- |

**Figure S10A.** Boxplots illustrating the proportions of various immune and stromal cell types across samples with favorable and unfavorable prognosis for Stage IV CRC patients.

|  | 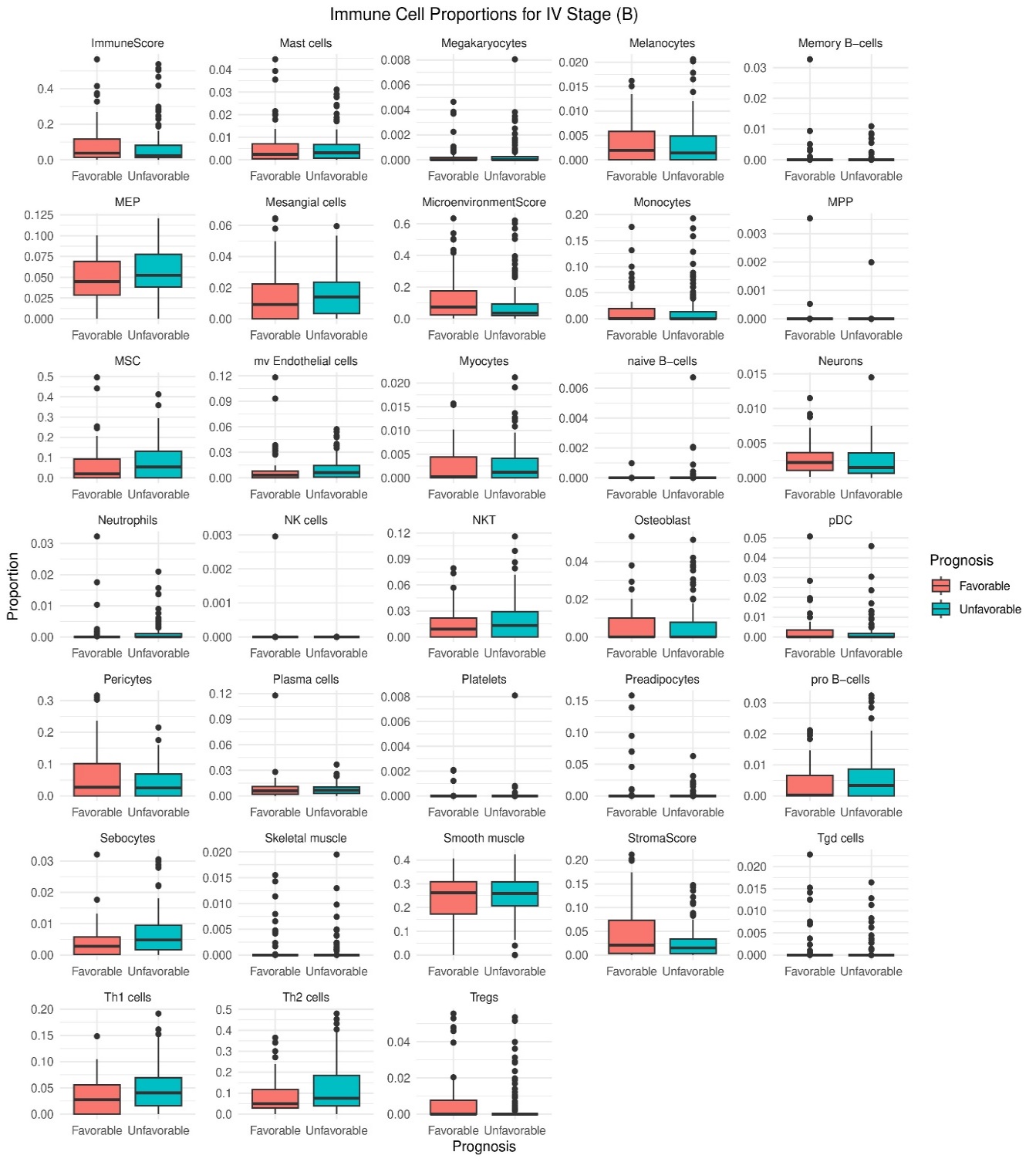 |
| --- | --- |

**Figure S10B.** Boxplots illustrating the proportions of various immune and stromal cell types across samples with favorable and unfavorable prognosis for Stage IV CRC patients.

**Table S12.** Differences in the proportions of cell types in primary tumor tissue for Stage II/III CRC patients stratified by prognosis. Raw p-values calculated with t-test and adjusted by the Benjamini-Hochberg method p-values are shown.

| **CellType** | **p-value** | **padj** |
| --- | --- | --- |
| Astrocytes | 3,1E-06 | 2,1E-04 |
| MEP | 1,7E-05 | 5,6E-04 |
| Chondrocytes | 1,5E-04 | 2,7E-03 |
| StromaScore | 1,6E-04 | 2,7E-03 |
| CLP | 8,9E-04 | 7,1E-03 |
| Endothelial cells | 8,0E-04 | 7,1E-03 |
| Fibroblasts | 6,9E-04 | 7,1E-03 |
| Osteoblast | 9,6E-04 | 7,1E-03 |
| Pericytes | 7,7E-04 | 7,1E-03 |
| Monocytes | 1,5E-03 | 1,0E-02 |
| MicroenvironmentScore | 3,5E-03 | 2,2E-02 |
| NKT | 4,3E-03 | 2,4E-02 |
| pDC | 6,2E-03 | 3,2E-02 |
| ly Endothelial cells | 7,5E-03 | 3,6E-02 |
| HSC | 9,1E-03 | 4,0E-02 |
| Megakaryocytes | 9,5E-03 | 4,0E-02 |
| Th2 cells | 1,2E-02 | 4,6E-02 |
| Melanocytes | 1,7E-02 | 6,3E-02 |
| Erythrocytes | 2,0E-02 | 7,1E-02 |
| iDC | 2,5E-02 | 8,5E-02 |
| CD8+ T-cells | 2,8E-02 | 8,5E-02 |
| Keratinocytes | 2,8E-02 | 8,5E-02 |
| Memory B-cells | 3,0E-02 | 8,7E-02 |
| mv Endothelial cells | 3,4E-02 | 9,5E-02 |
| CD4+ naive T-cells | 5,2E-02 | 1,4E-01 |
| Mesangial cells | 5,4E-02 | 1,4E-01 |
| Plasma cells | 6,0E-02 | 1,5E-01 |
| Adipocytes | 1,4E-01 | 2,7E-01 |
| B-cells | 1,4E-01 | 2,7E-01 |
| CD4+ Tcm | 1,3E-01 | 2,7E-01 |
| DC | 1,5E-01 | 2,7E-01 |
| ImmuneScore | 1,4E-01 | 2,7E-01 |
| Macrophages | 1,4E-01 | 2,7E-01 |
| Macrophages M1 | 1,4E-01 | 2,7E-01 |
| Macrophages M2 | 1,2E-01 | 2,7E-01 |
| aDC | 1,3E-01 | 2,7E-01 |
| naive B-cells | 1,3E-01 | 2,7E-01 |
| MSC | 1,8E-01 | 3,2E-01 |
| CD8+ Tem | 2,4E-01 | 4,1E-01 |
| Neurons | 2,6E-01 | 4,3E-01 |
| Neutrophils | 3,0E-01 | 4,9E-01 |
| CD4+ T-cells | 3,1E-01 | 5,0E-01 |
| MPP | 3,2E-01 | 5,0E-01 |
| Sebocytes | 3,4E-01 | 5,2E-01 |
| Hepatocytes | 3,8E-01 | 5,6E-01 |
| cDC | 4,5E-01 | 6,6E-01 |
| Epithelial cells | 4,9E-01 | 6,9E-01 |
| Class-switched memory B-cells | 5,0E-01 | 6,9E-01 |
| CD8+ naive T-cells | 5,2E-01 | 7,1E-01 |
| CD8+ Tcm | 5,5E-01 | 7,3E-01 |
| Basophils | 6,1E-01 | 7,7E-01 |
| CD4+ memory T-cells | 6,0E-01 | 7,7E-01 |
| Eosinophils | 6,0E-01 | 7,7E-01 |
| CMP | 6,4E-01 | 8,0E-01 |
| CD4+ Tem | 8,8E-01 | 9,5E-01 |
| Mast cells | 8,2E-01 | 9,5E-01 |
| Myocytes | 8,7E-01 | 9,5E-01 |
| NK cells | 8,7E-01 | 9,5E-01 |
| Skeletal muscle | 8,9E-01 | 9,5E-01 |
| Smooth muscle | 8,9E-01 | 9,5E-01 |
| Tgd cells | 7,8E-01 | 9,5E-01 |
| Th1 cells | 8,5E-01 | 9,5E-01 |
| Tregs | 8,5E-01 | 9,5E-01 |
| GMP | 9,3E-01 | 9,7E-01 |
| pro B-cells | 9,6E-01 | 9,9E-01 |
| Platelets | 9,9E-01 | 9,9E-01 |
| Preadipocytes | 9,7E-01 | 9,9E-01 |

**Table S13.** Differences in the proportions of cell types in metastatic tissue for Stage IV CRC patients stratified by prognosis. Raw p-values calculated with t-test and adjusted by the Benjamini-Hochberg method p-values are shown.

| **CellType** | **p-value** | **padj** |
| --- | --- | --- |
| Keratinocytes | 7,7E-05 | 5,1E-03 |
| Epithelial cells | 4,6E-04 | 1,5E-02 |
| Th1 cells | 3,9E-03 | 8,8E-02 |
| Sebocytes | 8,8E-03 | 1,5E-01 |
| Fibroblasts | 2,4E-02 | 2,3E-01 |
| StromaScore | 2,2E-02 | 2,3E-01 |
| Th2 cells | 2,0E-02 | 2,3E-01 |
| Hepatocytes | 3,7E-02 | 2,7E-01 |
| MEP | 3,3E-02 | 2,7E-01 |
| Adipocytes | 5,3E-02 | 3,6E-01 |
| Preadipocytes | 6,2E-02 | 3,8E-01 |
| CD4+ naive T-cells | 9,5E-02 | 4,1E-01 |
| CD8+ T-cells | 8,1E-02 | 4,1E-01 |
| CLP | 8,3E-02 | 4,1E-01 |
| CMP | 1,1E-01 | 4,1E-01 |
| Chondrocytes | 9,5E-02 | 4,1E-01 |
| DC | 1,3E-01 | 4,1E-01 |
| MicroenvironmentScore | 1,4E-01 | 4,1E-01 |
| Pericytes | 1,2E-01 | 4,1E-01 |
| Tregs | 1,2E-01 | 4,1E-01 |
| cDC | 1,3E-01 | 4,1E-01 |
| pro B-cells | 1,4E-01 | 4,1E-01 |
| HSC | 1,5E-01 | 4,3E-01 |
| Tgd cells | 1,6E-01 | 4,3E-01 |
| naive B-cells | 1,6E-01 | 4,3E-01 |
| Basophils | 1,9E-01 | 4,4E-01 |
| Neurons | 1,8E-01 | 4,4E-01 |
| aDC | 2,0E-01 | 4,4E-01 |
| iDC | 1,8E-01 | 4,4E-01 |
| ly Endothelial cells | 1,9E-01 | 4,4E-01 |
| B-cells | 2,5E-01 | 5,1E-01 |
| CD8+ naive T-cells | 2,5E-01 | 5,1E-01 |
| Class-switched memory B-cells | 2,6E-01 | 5,1E-01 |
| Smooth muscle | 2,6E-01 | 5,1E-01 |
| Skeletal muscle | 2,7E-01 | 5,2E-01 |
| CD8+ Tem | 2,9E-01 | 5,3E-01 |
| CD8+ Tcm | 3,3E-01 | 5,5E-01 |
| Erythrocytes | 3,2E-01 | 5,5E-01 |
| NK cells | 3,2E-01 | 5,5E-01 |
| pDC | 3,1E-01 | 5,5E-01 |
| ImmuneScore | 3,5E-01 | 5,7E-01 |
| CD4+ Tcm | 3,8E-01 | 5,9E-01 |
| Memory B-cells | 3,8E-01 | 5,9E-01 |
| CD4+ T-cells | 4,0E-01 | 6,0E-01 |
| MPP | 4,2E-01 | 6,2E-01 |
| NKT | 4,4E-01 | 6,5E-01 |
| Plasma cells | 4,6E-01 | 6,6E-01 |
| CD4+ memory T-cells | 5,1E-01 | 7,1E-01 |
| MSC | 5,8E-01 | 7,8E-01 |
| Monocytes | 5,8E-01 | 7,8E-01 |
| Mast cells | 6,2E-01 | 8,1E-01 |
| Macrophages M1 | 6,6E-01 | 8,5E-01 |
| Myocytes | 6,7E-01 | 8,5E-01 |
| Astrocytes | 8,3E-01 | 9,5E-01 |
| Eosinophils | 8,0E-01 | 9,5E-01 |
| GMP | 8,5E-01 | 9,5E-01 |
| Megakaryocytes | 8,2E-01 | 9,5E-01 |
| Mesangial cells | 8,4E-01 | 9,5E-01 |
| Neutrophils | 8,2E-01 | 9,5E-01 |
| mv Endothelial cells | 8,3E-01 | 9,5E-01 |
| Osteoblast | 8,7E-01 | 9,6E-01 |
| CD4+ Tem | 9,5E-01 | 9,7E-01 |
| Endothelial cells | 9,6E-01 | 9,7E-01 |
| Macrophages | 9,1E-01 | 9,7E-01 |
| Macrophages M2 | 9,7E-01 | 9,7E-01 |
| Melanocytes | 9,7E-01 | 9,7E-01 |
| Platelets | 9,6E-01 | 9,7E-01 |
